# Role of Nanoparticle-Conjugates and Nanotheranostics in Abrogating Oxidative Stress and Ameliorating Neuroinflammation

**DOI:** 10.3390/antiox12101877

**Published:** 2023-10-18

**Authors:** Tapan A. Patel, Bhavesh D. Kevadiya, Neha Bajwa, Preet Amol Singh, Hong Zheng, Annet Kirabo, Yu-Long Li, Kaushik P. Patel

**Affiliations:** 1 Department of Cellular and Integrative Physiology, University of Nebraska Medical Center (UNMC), Omaha, NE 68198, USA; kpatel@unmc.edu; 2 Department of Pharmacology and Experimental Neuroscience, College of Medicine, University of Nebraska Medical Center (UNMC), Omaha, NE 68198, USA; bbhaveshpatel@gmail.com; 3 University Institute of Pharma Sciences (UIPS), Chandigarh University, Mohali 140413, Punjab, India; nehabajwa2765@gmail.com (N.B.); preetnabha67@gmail.com (P.A.S.); 4 Division of Basic Biomedical Sciences, Sanford School of Medicine of the University of South Dakota, Vermillion, SD 57069, USA; hong.zheng@usd.edu; 5 Division of Clinical Pharmacology, Vanderbilt University Medical Center, Nashville, TN 37232, USA; annet.kirabo@vanderbilt.edu; 6 Department of Emergency Medicine, University of Nebraska Medical Center (UNMC), Omaha, NE 68198, USA; yulongli@unmc.edu

**Keywords:** nanoparticle-conjugates, nanotheranostics, oxidative stress, neurodegeneration

## Abstract

Oxidative stress is a deteriorating condition that arises due to an imbalance between the reactive oxygen species and the antioxidant system or defense of the body. The key reasons for the development of such conditions are malfunctioning of various cell organelles, such as mitochondria, endoplasmic reticulum, and Golgi complex, as well as physical and mental disturbances. The nervous system has a relatively high utilization of oxygen, thus making it particularly vulnerable to oxidative stress, which eventually leads to neuronal atrophy and death. This advances the development of neuroinflammation and neurodegeneration-associated disorders such as Alzheimer’s disease, Parkinson’s disease, epilepsy, dementia, and other memory disorders. It is imperative to treat such conditions as early as possible before they worsen and progress to irreversible damage. Oxidative damage can be negated by two mechanisms: improving the cellular defense system or providing exogenous antioxidants. Natural antioxidants can normally handle such oxidative stress, but they have limited efficacy. The valuable features of nanoparticles and/or nanomaterials, in combination with antioxidant features, offer innovative nanotheranostic tools as potential therapeutic modalities. Hence, this review aims to represent novel therapeutic approaches like utilizing nanoparticles with antioxidant properties and nanotheranostics as delivery systems for potential therapeutic applications in various neuroinflammation- and neurodegeneration-associated disease conditions.

## 1. Introduction

Oxidative stress refers to an inequity between the creation of reactive oxygen species (ROS) or free radicals and the protective antioxidant system [1]. The three most prevalent free radicals are hydroxyl radical (OH·), superoxide (O_2_·−), and hydrogen peroxide (H_2_O_2_). When superoxide (O_2_·−) is excessively produced, it reacts with nitric oxide (NO) to create peroxynitrite (ONOO), which is a reactive nitrogen species (RNS). Peroxynitrite can also produce reactive oxygen species (ROS) and result in apoptotic cell death [2]. During oxidative damage, there is an increased presence of both ROS and reactive nitrogen species (RNS). These molecules can directly oxidize various lipids and proteins (both cytoplasmic and membranous) in the nervous system, leading to cell and tissue deterioration and loss of function [3,4,5]. The generation of free radicals is associated with many critical pathogenic illnesses, including neurodegenerative disorders. In particular, the generation of free radicals is strongly associated with the progression of neurological disorders, including Alzheimer’s disease (AD), Parkinson’s disease (PD), and other neurodegenerative diseases, as well as many critical pathogenic illnesses, such as chronic inflammatory diseases, chronic heart failure, myocardial infarction, and multiple sclerosis. The generation of free radicals plays a crucial role in all these conditions. Free radicals have been demonstrated to disrupt the proper folding of proteins, impair the function of glial cells, cause mitochondrial dysfunction, and ultimately lead to cellular apoptosis [6,7]. Neurodegenerative disorders, such as dementia and loss of cognition, are characterized by the atrophy of nervous tissue, the formation of a large number of plaques, and the presence of neurofibrillary tangles [8,9]. In AD, the hallmark event is the aggregation of misfolded Tau protein and amyloid β proteins. In PD and Huntington’s disease (HD), respectively, the accumulation of α-synuclein and the mutant Huntington protein is observed [10,11].

This review aims to systematically discuss the impact of oxidative damage on various cell organelles and their association with the progressive loss of neurons, then discuss the different natural antioxidants and their limitations in averting damage due to ROS. Finally, the bulk of the review will focus on discussing the formulation and use of novel therapeutic approaches to prevent neurodegeneration by nullifying the effects of free radical-mediated oxidative damage in various neural tissues.

## 2. Oxidative Stress, Neurodegeneration, and Neurodegenerative Disorders

Oxidative stress enhances the production of free radicals that attack various types of neural cells and cause loss of structure and/or function of neurons. ROS or free radicals can disrupt proper protein folding and increase misfolded proteins that lead to neural cells with altered structure and function. ROS also leads to the malfunctioning of neuronal cells and their various cell organelles, such as mitochondria, endoplasmic reticulum, and the Golgi complex [6,7], as detailed in the following sections. 

### 2.1. Oxidative Stress and Its Impact on the Structure and Function of the Nervous System

Oxidative stress has a direct impact on the structure and function of various cells of the nervous system. ROS contributes to neuronal damage by attacking different macromolecules like lipids, proteins, and nucleic acids, affecting their molecular function and altering basic physiological function [12]. ROS production mediated by aggregation Amyloid β was documented to enhance lipid peroxidation, affecting the permeability of the membrane and stimulating excitotoxicity due to an increased influx of calcium (Ca^2+^). As a result of altered membrane permeability, there is a significant change in the normal physiology of synapse, resulting in altered neuronal transmission and consequently leading to impaired cognitive functions, learning, and memory [13,14,15,16].

### 2.2. Mitochondrial Dysfunction and Neurodegeneration

The mitochondria are the key organelles in neurons that provide the bulk of energy to power the multitude of neurons that make up the brain and spinal cord (Central Nervous System—CNS). They exhibit a key role in buffering presynaptic calcium signals and thus modulating neurotransmission [17]. Mitochondria possess their own molecular machinery (DNA) for synthesizing RNA and specific proteins. ROS production affects mitochondrial DNA and leads to mutation or damage, which alters the normal functioning of mitochondria, such as the biosynthesis of electron transport chain (ETC) proteins [18,19]. The respiratory chain or ETC of mitochondria consists of five complexes (complexes I, II, III, IV, and V). These complexes are responsible for generating adenosine triphosphate (ATP) from adenosine diphosphate (ADP). Mitochondria are involved in generating free radicals from the activity of the ETC and are the key target of ROS. Mitochondria produces the majority of ROS from complexes I and III [20,21]. 

It is interesting to note that mitochondrial dysfunction is most observed in neurodegenerative conditions and disorders such as AD and PD. [22,23]. Altered activities of various enzymes (pyruvate dehydrogenase and isocitrate dehydrogenase) involved in various energetic pathways, such as the tricarboxylic acid cycle, were observed in the brains of patients with AD [24]. Reduced capacitance, selective loss of neurons in substantia nigra, and motor deficits were observed in mitochondrial transcription factor A-deficient mice [25]. Mitochondrial dysfunction leads to aggregation of pTau due to the elevation of free radicals and calcium ions, as well as also due to deficiency of superoxide dismutase-2 [26,27]. Normally, various antioxidant systems of cells play an important role in reducing oxidative damage. However, significantly reduced levels of non-enzymatic antioxidants such as glutathione and other antioxidant enzymes have been documented in AD and mild cognitive impairment (MCI) patients [28,29]. 

In amyotrophic lateral sclerosis (ALS) (motor neuron disease), anomalies have been reported in mitochondrial respiratory chain enzymes, mitochondrial assembly, as well as mitochondrial cell death proteins. It has been demonstrated that oxidative damage leads to mitochondrial dysfunction, loss of motor neurons, and, finally, neurodegeneration, leading to ALS [30,31]. An increased level of a lipid peroxidation product, malondialdehyde (MDA), was noted in the brains of AD and MCI patients due to oxidative stress [32]. Apart from generating free radicals, mitochondria are highly vulnerable to oxidative imbalance generated by other factors, such as stress, environmental factors, and age. Mitochondrial dysfunction is also observed in the pathologies of AD [33] (Table 1). ROS causes lipid peroxidation of the mitochondrial inner membrane and leads to proton leakage. Such alteration causes mitochondrial dysfunction by impairing different biochemical activities of proteins (transporters and respiratory enzymes). Calcium homeostasis is one of the key regulators for normal neuronal functioning. ROS alters the equilibrium of Ca^2+^ ions in the mitochondria of neurons via membrane alteration. Alteration in Ca^2+^ ions causes alteration in the potential of mitochondria, which in turn produces more superoxides. Excessive overload of Ca++ ions in mitochondria promotes neuronal loss or apoptosis [20,34]. Interaction of free radicals, mitochondrial dysfunction, and altered calcium signaling in neurons and astrocytes has been implicated in different neurodegenerative diseases, including AD [35], as well as in neurons and astrocytes, leading to a loss of neuronal structure and function [36]. In transgenic AD mice models and AD patients, altered expression of disrupted-in-schizophrenia 1 (DISC1), a mitophagy receptor, has been noted. Also, DISC1 is responsible for mitophagy after binding to the microtubule-associated proteins 1A/1B light chain 3 [37]. Oxidative damage and malfunctioning of mitochondria have also been found to be associated with the localization of APOE4 (E4 variant of apolipoprotein E) to the mitochondria and modulating the expression of mitochondrial genes related to the neurodegeneration [38,39]. Generation of ROS and release of inflammatory cytokines (such as IL-6, IL-1β, and TNFα) are closely associated with axonal damage in cerebellar cultures. It is of interest to note that such cytokines are increased after the deposition of amyloid plaques in AD [40,41]. BACE1, a rate-limiting enzyme involved in generating amyloid-β peptides (Aβ) in AD, is elevated due to the depletion of nuclear factor erythroid-derived 2, which represses the expression of BACE1 [42].

Association between increased ROS and mutation in genes coding for PTEN-induced kinase 1 (PINK1), Parkin, α-synuclein, and DJ-1 have been well documented in PD. Alteration of these proteins and aggregation of α-synuclein contributes to the development and progression of PD and neuronal loss [43,44]. Furthermore, impaired metabolism of dopamine has been observed to generate free radicals (via the Fenton mechanism) in different neurodegenerative diseases, such as AD, PD, and dementia. ROS-induced altered levels of glutathione were observed in neurons of the transgenic model. The consequence of such a prolonged reduction in glutathione in dopaminergic neurons leads to impairment of mitochondrial complex I activity via thiol oxidation [45,46,47,48]. Aggregation of α-synuclein was observed in neurons with DJ-1 mutation of PD patients. A lack of DJ-1 or PINK1 showed enhanced ROS in the mitochondria of neurons and their death [49,50,51]. Enhanced levels of NLRP3-associated inflammasome in activated microglia have been observed in activated microglia and neurons of neurodegenerative disorders. Such enhanced NLRP3 triggers the release of inflammatory cytokines (IL-1β and IL-18). These cytokines are responsible for neuronal atrophy and death [52,53]. Damaged mitochondria generate excessive ROS, and the released mtDNA, in turn, enhances NLRP3. 1-methyl-4-phenyl-1,2,3,6-tetrahydropyridine (MPTP)-induced mouse model of PD showed stimulation of NLRP3 and a loss of dopaminergic neurons followed by motor deficit, whereas a lack of NLRP3 in MPTP-treated mice showed reduced neurodegeneration and inhibition of cytokine production [54]. On the other hand, elevated levels of ROS enhanced α-synuclein aggregates in a transgenic model of PD due to haplodeficiency for SOD2 [55]. Treating mouse astrocytes with α-synuclein showed enhanced expression of NLRP3, IL-1β, and caspase-1 [56]. Microglia with enhanced NLRP3 inflammasome showed significant neuronal death [54]. Enhanced levels of caspase-1 produce IL-1β and IL-18 from their inactive state, which are active inflammatory cytokines. Elevated levels of IL-1β and IL-18 showed pyroptosis (an inflammatory form of programmed cell death) in many neurodegenerative diseases, such as AD, ALS, HD, PD, and multiple sclerosis [57,58].

Hence, mitochondrial dysfunction can be a key cause for generating ROS as well as a consequence of oxidative stress.

### 2.3. Endoplasmic Reticulum (ER) Stress and Neurodegeneration

The endoplasmic reticulum, the dynamic organelle of the cell, plays many critical roles, such as protein synthesis, calcium storage, and metabolism of macromolecules (lipids and proteins). Under normal physiological conditions, ER maintains normal protein folding and trafficking. Such diverse functions are performed by its key domains, i.e., tubules, and the nuclear envelope [59,60]. Chaperones in the cytosol and the ER maintain the folding of newly synthesized proteins, whereas cellular quality control mechanisms recognize abnormally misfolded proteins and accelerate their degradation via the proteasome and autophagy pathways. However, during oxidative imbalance, cellular quality control mechanisms get severely affected and lead to the generation of misfolded proteins, resulting in the loss of structure and function of neurons [61,62]. Disturbances in the structure and function of the ER due to free radical-associated stress or any environmental stress can lead to ER stress, which in turn enhances abnormal and unfolded protein aggregation. Accumulating such abnormal unfolded proteins and changes in calcium homeostasis within the ER can also contribute as a key factor for ER stress [63,64,65]. The protein folding process depends on redox homeostasis. Hence, oxidative damage can affect the process of protein folding and augment the generation of misfolded proteins, leading to ER stress. Several studies have shown that alteration in the biosynthesis of proteins and inhibition of the formation of disulfide bridges can generate misfolded proteins. Various studies have demonstrated that changes in protein biosynthesis and inhibition of disulfide bridge formation can contribute to the generation of misfolded proteins [66,67,68,69]. Disulfide bridges are important for the correct folding and stabilization of proteins, and their absence or incorrect formation can lead to protein misfolding and aggregation. In normal physiological circumstances, the ER possesses a highly specific quality control mechanism to remove the proteins that are misfolded [67,70,71]. During oxidative imbalance, ER homeostasis can be compromised, which can lead to the accumulation of misfolded proteins and the generation of ER stress (ERS). ERS can reduce the rate of transcription and translation and affects many signaling pathways, leading to various disease conditions [72]. Also, the accumulation of misfolded proteins in the brain is observed in many pathological conditions of neurodegenerations [73] (Table 1). Hence, long-term ER stress can be a cause of neurodegenerative disorders.

### 2.4. Golgi Apparatus and Neurodegeneration

The GC is very well organized while surrounding the nucleus in neurons [74,75]. The arrangement of GC in the neuron is important for dendrite formation in the adult brain [76]. The GC of neurons is essential for supporting axodendritic polarity [77]. The tubular structures of GC contain specific glycosylation enzymes that are involved in the sorting and post-translational modification of proteins [78]. Endothelial Reticulum-to-Golgi transport is necessary for dendritic growth [79]. Overall, the GC plays a central regulatory role in maintaining cellular homeostasis. Enhanced free radicals cause structural rearrangements in the GC known as Golgi fragmentation. These structural rearrangements in GC are associated with a loss of neuronal structure and the development of neurodegenerative diseases. Fragmentation of the Golgi apparatus has been observed in several neurodegenerative diseases [80]. Golgi fragmentation is also associated with the inhibition of ER-Golgi trafficking [81]. In neurological disorders, such as AD, Golgi fragmentation is associated with the serine/threonine kinase CDK5, which phosphorylates specific proteins such as GM130 and GRASP65 [82,83] of the Golgi matrix. Phosphorylation of the microtubule-binding protein, Tau, by CDK5 leads to Golgi fragmentation in AD [84,85]. Golgi fragmentation may also affect the physiology and function of neurons in neurodegenerative disease as well as alters the transport processes (protein trafficking) in axons and synapses [86,87,88] (Table 1). Hence, oxidative stress may lead to Golgi fragmentation, which in turn affects the structure and function of neurons and the nervous system, ultimately leading to neurodegeneration.

**Table 1:** List of cellular organelle damages due to oxidative stress and related pathways.

Cellular Organelle Damage	Model System	Consequence	References
Mitochondrial dysfunction	Brain of patients suffering from AD	ROS production and amyloid plaques	[89,90]
	Fibroblasts of patients suffering from X-linked adrenoleukodystrophy (a neurodegenerative condition)	Dissipation of mitochondrial inner membrane potential, ATP drop, and enhanced oxidative modifications in cyclophilin D protein	[91]
	Blood of PD patients	Higher level of ROS and diminished cytochrome c oxidase activity	[92]
	Patients suffering from AD	Oxidative damage	[93]
	STHdhQ7 and STHdhQ111 striatal model of HD (in vitro); human HD striata and skin fibroblasts	Mitochondrial DNA lesions and ROS; decreases in respiratory capacity	[94]
	Lewis rats, with parkinsonism-like symptoms	Mitochondrial dysfunction and its ultrastructural damage due to oxidative and nitrosative stress as well as translocation of Bim and Bax from cytosol to mitochondria	[95]
ER stress	The hippocampal pyramidal cells and the frontal cortex of AD brains	Increased phosphorylation of PERK and eIF2α	[96]
	Striatal cell line (expressing pathogenic huntingtin); brain of HD model mice; prion-diseased mice	Elevated phosphorylation of eIF2α	[97,98]
	PARK20 (Parkinson disease 20) fibroblasts	Production of ROS as well as activation of the Unfolded Protein Response-associated PERK/eIF2α/ATF4/CHOP pathway	[99]
	Human melanoma cells	Increased ROS levels	[100]
	Vascular smooth muscle cells (VSMCs) in spontaneously hypertensive rat (SHR)	Enhanced ROS levels due to overexpression of Nox1 and Nox4	[101]
	Rat cortical neurons	Spliced ATF6; elevated levels of CHOP, PERK, p-eIF2α, and ROS	[102]
Golgi Fragmentation	AD model (neurons of the hippocampus from transgenic mouse and BV-2 cell)	Depletion of coat protein I (COPI) expression	[103]
	Primary hippocampal cells and HeLa cells	Reduction in the number of cisternal membranes per Golgi stack due to H_2_O_2_ treatment	[104,105]
	Primary cerebrocortical cells	Oxidative and/or nitrosative insults	[106]
	Neurons of amyotrophic lateral sclerosis animal model (SOD1-ALS mouse model)	Mutation of SOD1	[86]
	Mouse neuroblastoma N2a cells and cultured neurons	Oxygen–glucose deprivation/reperfusion (OGDR) insult and ROS production	[107,108,109]
	Primary cultured hippocampal neurons and primary cortical neurons	Increased cyclin-dependent kinase 5 (Cdk5) activity via oxidative stress	[110,111]

## 3. Natural Antioxidants, Their Importance, and Limitations

Natural antioxidants are naturally occurring biomolecules that can reduce free radicals and maintain a balance between ROS and the antioxidant system. Such antioxidants may be divided into two types: (i) enzymatic and (ii) non-enzymatic antioxidants. Generally, enzymatic antioxidants transform free radicals into less-reactive hydrogen peroxide and, ultimately, to water. This reaction requires the presence of cofactors such as manganese, copper, and zinc for an array of processes, while other non-enzymatic antioxidants disrupt a chain reaction of free radicals. Such non-enzymatic antioxidants include polyphenols, carotenoids, and vitamins C and E [112,113].

### 3.1. Natural Antioxidants and Their Importance

Many naturally occurring antioxidant compounds are found in fruits, vegetables, and cereals. Polyphenols are one of them. Certain fruits such as apples, grapes, oranges, berries, and cherries contain polyphenols in significant amounts. These polyphenols are considered as secondary metabolites of plants and are commonly involved in defense against pathogens [114,115,116]. Long-term consumption of polyphenol-rich diets provides protection against various diseases such as cardiovascular diseases, diabetes, osteoporosis, and neurodegenerative diseases, as well as the development of cancers known to have increased free radicals [114,115,117]. Polyphenols can be classified into phenolic acids, flavonoids, and lignans [115]. Polyphenols present in the tea are thought to be involved in reducing blood pressure due to their antioxidant activity as well as their positive effect on the functions of endothelial cells [116]. Fruits and vegetables contain a broad variety of antioxidants, such as vitamins and polyphenols. Polyphenols are polyhydroxylated phytochemicals, which possess two key classes, i.e., flavonoids and phenolic acids. Flavonoids comprise a large group of polyphenols divided into several subclasses, such as flavones, flavanones, and flavanols [118,119]. Several fruits, such as apples, grapes, blueberries, blackberries, raspberries, and strawberries, are rich in polyphenolic compounds. Anthocyanins are key polyphenolic compounds present in colored fruits, which have beneficial effects against various diseases, such as pancreatic cancer [120,121]. Polyphenolic compounds such as Silymarin exhibited promising action in preventing and treating several diseases, such as neurodegenerative diseases, and oxidative damage due to physical exercise [122,123,124,125].

Carotenoids are efficient scavengers of singlet oxygen and other reactive oxygen species [126]. Carotenoids play an important role, such as boosting antioxidants and immunity [127]. Several carotenoids, such as astaxanthin, fucoxanthin, and lutein, are powerful antioxidants as well as anti-inflammatory agents [128,129,130,131] in cardiovascular diseases [132] and neurodegenerative disorders [133,134]. Carotenoids can effectively neutralize free radicals and exhibit different levels of antioxidant activities because of the presence of highly polarized functional groups [134]. In a study involving participants with type 1 diabetes, it was observed that a greater intake of fruits, vegetables, and carotenoids was associated with higher levels of carotenoids in serum with no moderation of glucose levels or oxidative injury [135]. However, Zeaxanthin, a carotenoid, has been proven to avert diabetic retinal abnormalities and retinal dysfunction by regulating oxidative damage [136]. In a focal brain ischemia rat model, β-carotene administration significantly reduced nuclear factor kappa B in the brain while maintaining the histological architecture of brain tissues. β-carotene also significantly suppressed Bcl-2-associated X protein and caspase-3 expression, suggesting a neuroprotective action [137].

Vitamins are biological substances that are required for the regular functions of cells, as well as for the growth and development of the body. Among all vitamins, vitamins C and E exhibit significant antioxidative effects by controlling oxidative damage. Vitamin C and E improved antioxidant enzymes and reduced free radicals in a rat model with repetitive loading exercise as well as aging [138]. Employees of a power plant where they are routinely exposed to extremely low-frequency electromagnetic fields, which is known to cause oxidative damage, showed a declining trend of lipid peroxidation with an enhancement in the level of antioxidant enzymes after supplementation of vitamin C (1000 mg) and E (400 units) every day for 90 days [139]. Vitamin E is a known antioxidant and is ubiquitously present in vegetables. It has been shown to have various therapeutic applications [140]. Chronic administration of Vitamin E to Phosphatase and tensin homolog (PTEN)-induced kinase 1 knockout (PINK1^−/−^) mice showed full restoration of corticostriatal synaptic plasticity. Thus, it has been suggested to strongly protect against neurological diseases such as PD [141]. A vitamin E-rich diet may minimize the risk of PD. Vitamin E shields our body from the destructive effects of free radicals by neutralizing various them [142,143]. Vitamin C prevents age-induced oxidative damage in hippocampal neurons by controlling free radical damage, as demonstrated in an in vitro study [144]. A clinical study has documented the protective effect of combined administration of vitamin E and C to delay the onset of neurodegenerative diseases (AD) in older people [145]. It has also been exhibited that a co-supplementation of vitamin E with omega-3 fatty acids can avert lipid peroxidation in PD patients. Also, this co-supplementation showed a decline in high-sensitivity C-reactive protein and increased levels of glutathione and total antioxidant capacity in PD patients [146].

### 3.2. Limitations of Natural Antioxidants and the Need for Novel Therapeutics

It has been well documented that an enormous amount of antioxidant compounds are present in nature, and they have protective action against many diseases. However, such compounds have limitations, such as low shelf life and trivial amounts of antioxidants. Apart from these limitations, even such antioxidant compounds also have low bioavailability and poor water solubility. Further, the extraction and long-term storage of pure and active compounds are very difficult and tedious. Furthermore, the levels of polyphenols and other antioxidants in these vegetables and fruits are inconsistent, and their levels vary with the seasons and particular environmental circumstances/conditions [122,147,148]. As a result of such variations in antioxidant compounds and their concentrations in vegetables and fruits, it becomes very difficult to advocate/decide the amount that should be ingested. As a consequence, it is necessary to determine the appropriate levels of such antioxidants for the therapeutic purpose; otherwise, they would have a negative impact on the health of a patient or even a healthy person. It has been very commonly presumed that the consumption of higher doses of such dietary supplements could become powerful disease-preventive and immune-boosting agents. However, in human studies, recently, two meta-analysis investigations with vitamins or micronutrient preparations documented that higher doses of antioxidant supplements may increase the frequency of cardiovascular diseases and cancer and result in a higher mortality rate [149,150]. Several antioxidants, such as carotenoids, have been isolated and studied from several microalgae, such as lutein from *Chlorella protothecoides*, β-carotene from *Dunaliella salina*, and astaxanthin from *Haematococcus pluvialis* [151]. Besides the negative impact of high doses of antioxidants on human health, the extraction of pure and active compounds from plants and several microalgae is very difficult due to the presence of rigid cell walls. Hence, the recovery of pure bioactive molecules from vegetables, fruits, and some microalgae poses a major challenge. To meet this challenge, there are several novel therapeutic approaches, which are discussed in the next section.

## 4. Role of Nanoparticles (NPs) as Reactive Oxygen Species (ROS) Scavengers and/or Modulators of Activities of Antioxidative Enzymes to Reduce Oxidative Stress in Neurodegeneration

Oxidative stress represents a prominent avenue through which cellular toxicity can manifest. Oxidative damage can cause uncontrolled cell signaling; morphological and physiological changes in cell and cellular organelles; and alterations in cell motility, apoptosis, cell death, and carcinogenesis [152,153]. Thus, the utility of antioxidants is indispensable in averting or, in most instances, mitigating the deleterious consequences induced by ROS. Notably, the hydroxyl radical exhibits pronounced reactivity toward a diverse array of biomolecules, encompassing proteins and nucleic acids, and boasts the loftiest one-electron reduction potential among physiologically significant ROS [154]. Antioxidants serve as the exclusive shield against the detrimental influence of hydroxyl radicals. Functioning as reducing agents, antioxidants engage in redox reactions by donating electrons or hydrogen atoms. By adhering to specific constraints, this enzymatic process facilitates the seamless execution of cellular functions while concurrently thwarting the oxidation of pivotal structural constituents and other indispensable elements [153,155,156].

A normal defensive reaction to stimuli, including infection, damage, and poisons, is inflammation. However, it has been shown that excessive and unchecked inflammation can cause illnesses, including heart disease, hepatitis, nephritis, and sluggish wound healing. It is well-accepted that oxidative imbalance and inflammation are closely related [157]. In an overly inflammatory state, ROS may exacerbate localized tissue damage and cause persistent inflammation that impairs blood–brain barrier permeability, resulting in affecting neural tissues [158,159]. Consequently, it has been postulated that employing a wide range of nanoparticles and their conjugates as antioxidants to scavenge ROS represents a viable therapeutic approach for managing diverse inflammatory conditions. Nonetheless, the clinical utility of these pharmacological agents is hampered by challenges such as limited absorption, chemical instability, and suboptimal efficacy, which impose constraints on their widespread momentum in the medicament of free radical-associated diseases [156]. Certain studies have demonstrated that conjugated or functionalized NPs can cross the blood–brain barrier (BBB) and exhibit their neuroprotective role against ROS-mediated neuronal toxicity and brain inflammation [160,161,162,163]. 

Advancements in nanotechnology medicine have paved the way for novel approaches to enable ROS clearance and the subsequent management of ROS-associated disorders. Through the utilization of diverse functional nanomaterials, including ceria, carbon, redox polymers, platinum, and polyphenol NPs, new avenues have emerged for effectively addressing ROS-related pathologies. The following sections detail the use of these various diverse functional nanomaterials and their utility in treating neurodegenerative diseases. 

### 4.1. Unique Properties of NPs

Nanoparticles (NPs) are tiny elemental entities in nanotechnology that exhibit unique properties due to their small size. They combine electrons, phonons, and photons at the nanometer level, leading to novel materials with distinct chemical, physical, and biological characteristics. For instance, in a 10 nm grain size, 14% to 27% of atoms can be found within 0.5–1.0 nm of a grain boundary [164]. Grain boundaries significantly impact material characteristics compared to microstructural metals and alloys. As the grain size decreases, disorderly interfaces and lattice defects increase, influencing material properties. The fraction of atoms near grain borders scales as 1/d with decreasing grain size (d). Ultrafine-grain materials are mainly influenced by interfacial rather than bulk characteristics [164]. NP reactivity is influenced by size, shape, and structure, along with other factors. Different reactions exist based on NP size, impacting properties like the melting point, optical and magnetic properties, reactivity, and conductivity. These shifts stem from quantum physics, where bulk characteristics result from the sum of all quantum forces on atoms [164,165,166].

**Figure 1:**
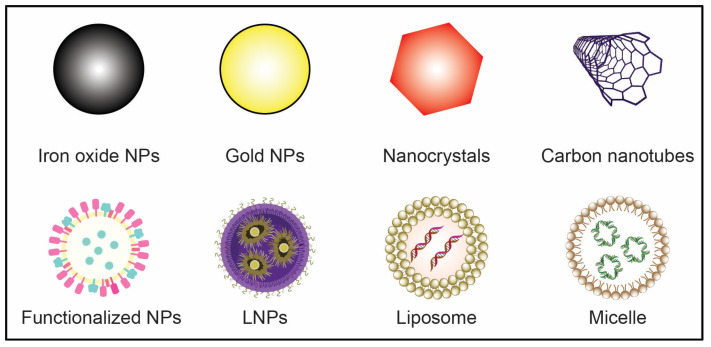
Different nanoparticles (NPs). Schematic representation of various functionalized NPs.

In general, quantum effects, increased surface area, and self-assembly are credited with giving nanomaterials their distinctive features. The behavior of matter at the nanoscale, especially at the lower end, can start to be dominated by quantum effects, which can affect how materials behave optically, electrically, and magnetically. It is believed that this is because the matter at the nanoscale no longer obeys Newtonian physics but rather quantum mechanics, which is explained by the quantum confinement, size effect, and density of states (DOS). Second, compared to the same mass of material generated in bulk form, nanoparticles have a considerably larger surface area. The proportion of surface atoms increases with decreasing particle size, increasing reactivity by increasing the number of active sites. In some instances, materials that are inert when created in their bulk form end up becoming reactive when produced in their nanoscale form. All nanomaterials, regardless of shape, including nanoparticles, nanocoatings, nanotubes, and nanowires, are affected by increasing surface areas [164]. Different NPs and their conjugates are mentioned in Figure 1. 

#### 4.1.1. Biological Properties

Due to the dynamic nature of many NPs, which obscures recognition by the reticuloendothelial system (RES) of the cells, they can be utilized to encapsulate a variety of medicines, proteins, enzymes, DNA, and siRNA. This allows for extended circulation times. NPs can enhance the pharmacokinetics, pharmacodynamics, and biodistribution of medicinal compounds due to their small size, which makes them better adapted to cross-cell membranes [167,168]. One of the most significant applications of NPs is in drug and gene delivery, which involves delivering therapeutically relevant DNA to in vitro or in vivo targets [169]. Due to their safety and simplicity of manipulation, polymeric nanoparticles have emerged as potential options among the suggested nanoparticles. Lipid nanoparticles have also been used for the delivery of genes because of their low toxicity and simplicity in surface functionalization. This enables the selective control of surface attributes like charge and hydrophobicity [170]. Additionally, Plasmid DNA (pDNA) was delivered using goldNPs chemically modified with primary and quaternary amine moieties. This method was more effective than traditional transfection agents for intracellular delivery [167,171,172].

#### 4.1.2. Biodistribution and Pharmacokinetics 

The particle size is one of the most essential elements defining the in vivo fate of NPs, such as retention in circulation, bypassing numerous biological barriers, and diffusion into tissues (Table 2). The particle size also dictates the uptake and removal by reticuloendothelial system (RES) cells, specifically in the liver and spleen. The size of the NPs will aid in their internalization by the target cells. According to the research, NPs smaller than 100 nm appear necessary for most in vivo applications because they can effectively diffuse through tissues and gain access to vascular fenestrations. The characteristics of the target cells may determine the preferred uptake mechanisms, which may be endocytosis, pinocytosis, or phagocytosis, as well as the ideal size for internalization [167].

#### 4.1.3. Cellular Internalization of NPs 

The plasma membrane of the cells selectively inhibits the uptake of therapeutic compounds with a molecular weight (MW) above 1 kDa, and cellular internalization is tightly restricted to the endocytic pathway [180]. Endocytosis is the process by which cationic nanoparticles enter cells after interacting with anionic cell membranes through electrostatic forces of attraction. Engulfment of nanoparticles in membrane invaginations to form endosomes, payload delivery by endosomes to various specialized vesicles, and delivery of the payload to various intracellular compartments are the three main steps of endocytosis of nanoparticles process [167,181,182,183]. It is important to note that 500 nm small latex particles were found to be internally processed by cells through an energy-dependent process, according to research on the impact of the particle size on the internalization pathway and subsequent intracellular fate in nonphagocytic B16 cells [184]. NPs’ size influences uptake efficiency. As the size increased from 50 to 500 nm, smaller particles were severely disrupted by microtubule disruption, while 500 nm-sized nanoparticles were unaffected. In a study on Caco-2 cell lines, 100 nm-sized PLGA NPs were taken up about 2.5 times more than 1 mm-sized and six times more than 10 mm microparticles [185]. The process of absorbing nanoparticles via mucosal and epithelial tissue is largely influenced by the size of the particles, with intracellular trafficking coming in second [186]. In an ex vivo canine carotid artery model, it was found that small nanoparticles (100 nm) had more than three times the arterial absorption of large nanoparticles (275 nm) [187,188]. The larger micron-particles were primarily localized in the epithelial lining, whereas the smaller nanoparticles could easily pass through the submucosal layers [167].

### 4.2. Application of Gold-Based NPs (AuNPs) and Their Antioxidant (Phytochemical) Conjugates in Oxidative Stress

Gold has a special reputation, both as a precious metal among its peers and as a noble biocompatible element, especially regarding its nanoparticle formulation [189]. This is due to its surface having a special effect that gives rise to different therapeutic activities [190]. Enhanced production of free radicals plays the most pivotal role in the pathology of several diseases [191], including some of the most chronic to acute ones. Here, antioxidants play a crucial role in relieving oxidative injury [192]. Unlike micro-organism-based AuNPs, plant-based or phytochemical-AuNP conjugates are environment-friendly, non-toxic, cost-effective, scalable, stable, and possess non-aggregation properties [193]. AuNPs (100 nm, spherical) synthesized from the rhizome of *Paeonia moutan* acted as an anti-inflammatory agent in the in vitro (murine microglial BV2 cells) model of PD [194]. On a similar note, Wang et al. designed AuNPs (20 nm, spherical) and stabilized them using flavonoids from the leaf extract of *Scutellaria barbata*, where the NPs were observed to be efficient in inhibiting aggregation of Aβ40, thereby acting as a novel β-amyloid inhibitor [195]. 

Inflammation is also known to result in oxidative damage [196]. Species like plumeria and others have been apprised due to their anti-inflammatory and wound-healing (where ROS have an important role to play) properties, respectively [197,198,199]. AuNPs conjugates synthesized from the leaf extract of *Euphrasia officinalis* showed various shapes, including spherical, hexagonal (with lattice fringes), and triangular. AuNPs significantly reduced the phosphorylation and degradation of inflammatory mediators (IκB-alpha) and inhibited the nuclear translocation of NF-κB p65, acting as an anti-inflammatory agent [200]. Similarly, AuNPs combined with epigallocatechin gallate and α-lipoic acid significantly accelerated cutaneous wound healing in mice models [201]. Furthermore, chronic inflammation has long been evidenced to be responsible for the initiation (and progression) of various diseases, such as malignancies [202], by reducing the body’s defense wall (i.e., antioxidants) against angiogenesis and metastasis [203]. Here too, AuNP conjugates have shown their unique pharmacotherapeutic properties, as apparent from the first-of-its-kind study. AuNP-silibinin (AuNP-Sb) conjugates (spherical, 163 ± 5 nm) were formulated and targeted to treat lung cancer cells. To their amazement, combining Sb with AuNP increased its efficacy ~5-fold when compared to its control [204]. Therefore, from the above studies, it is evident that phytochemical conjugates of AuNPs could be of potential therapeutic importance to alleviate inflammation and moderate ROS. 

The capacity of AuNPs as nanocarriers has received much attention for treating neural tissues. Gold nanoparticles contain numerous and specific properties, which make them suitable as nanocarriers for crossing the BBB. Their ability to be tailored into various shapes and positions, which they can combine with their low toxicity and compatibility with living systems, make them a compelling choice [205,206,207]. Moreover, gold nanoparticles and their combinations with other compounds can mimic the enzymatic activities of superoxide dismutase, oxidase, glucose oxidase, peroxidase, and catalase [206,207]. Specifically, in terms of exhibiting superoxide dismutase activity, which involves converting superoxide ions into hydrogen peroxide and oxygen, gold nanozymes operate similarly to natural enzymes [205]. In the process of catalase mimicking action, hydrogen peroxide reduces the Au^2+^ to Au^+^, leading to the production of protons and oxygen. To clear hydrogen peroxide, a second hydrogen peroxide reacts with oxygen vacancies, resulting in the oxidation of gold to gold dioxide and the production of water. AD is characterized by neuronal degeneration, commonly observed in the hippocampus region, entorhinal cortex, frontal cortex, and amygdala. Recent research has shown that gold nanozymes have the potential to mitigate mitochondrial dysfunction, which is a significant contributor to neurodegenerative diseases. Additionally, these nanozymes can help to combat acute oxidative damage and unwanted inflammation in the cortex and hippocampal regions of the brain [205,208]. A recent study on AD rat models involved an intracerebroventricular injection of okadaic acid. Then, they underwent 21 days of treatment with 20 nm AuNPs (dose: 2.5 mg/kg) every 2 days. Animals given only okadaic acid had higher levels of Tau phosphorylation in their cortex and hippocampus, while those given AuNP showed normal levels. AuNP therapy reduced elevated levels of nitrite, free radicals, and sulfhydryl in the brain caused by okadaic acid [209]. Additionally, it has been observed that this therapy improves the levels of catalase, glutathione, and superoxide dismutase, which are important antioxidant enzymes involved in cellular defense mechanisms [205,209,210].

Oxidative injury is a key factor for demyelination as well as neurodegeneration in multiple sclerosis [3,211]. It was demonstrated that AuNPs stabilized with glutathione could easily pass the BBB and prevent the aggregation of Aβ-42 fibrils without causing any side effects [212]. Oligodendrocyte Progenitor Cells (OPCs) in multiple sclerosis (MS) are unable to remyelinate because of the cellular stress they experience, which also causes bioenergetic processes to fail [205,213,214]. Oral administration of gold nanocrystals improved motor functions in cuprizone-treated animals. In vitro studies demonstrated enhanced oligodendrocyte maturation, increased expression of myelin differentiation markers, and upregulation of genes associated with myelin synthesis due to the gold nanocrystal treatment [215]. Gold nanocrystals have shown promise in treating multiple sclerosis (MS) by promoting remyelination and enhancing oligodendrocyte progenitor cell (OPC) differentiation. The mechanism of action involves a nanocatalytic process that utilizes NAD+ and NADH [205,215]. As a result, gold nanoparticles are extremely dependable in usage. Additionally, due to their versatility, they are a special material with a wide range of potential uses.

### 4.3. Protective Effect of Silver-Based NPs (AgNPs) and Their Antioxidant Conjugates in Oxidative Stress

Silver is considered second to gold when it comes to the precious list of metals. AgNPs also have remarkable characteristics, such as a wide surface area, excellent dispersion, and small size. These features of AgNPs aid in their wide biomedical applications as antioxidant, anti-inflammatory, antimicrobial, anticancer, and antidiabetic agents. The pathology of all of these is related to oxidative injury [216,217,218,219]. The synthesis of AgNPs from Tamarix articulata leaf extract showed potential anti-inflammatory and antimicrobial activity. The AgNPs obtained were spherical and exhibited antioxidant activity (DPPH scavenging: 68.23%, H_2_O_2_ reduction: 70.09%, ferric3+ reduction: 68.23%). They also demonstrated potent anti-inflammatory properties (inhibition of albumin denaturation: 73.19%, protease activity: 70.196%, membrane stability against heat: 74.16%, and hyposaline-induced hemolysis: 72.98%), as well as antimicrobial activity against five bacterial strains and one fungal strain, when compared to controls [220]. Similarly, Küp and associates synthesized AgNPs using leaf extract of *Aesculus hippocastanum,* which were spherical and had a size of 50 ± 5 nm. Moreover, these NPs exhibited strong antibacterial activity (against 8 Gram (+) ve and 6 Gram (−) ve bacteria) and antioxidant activity (DPPH scavenging (54.72% at 100 ppm), as well as superoxide radical scavenging activity (62.9%)). In addition, it was also noted that the resveratrol-conjugated AgNP had a capping effect, as it reduced the flank influence of resveratrol on normal tissues [221]. Therefore, from the above studies, it could be understood that AgNP (extract) conjugates could be of potential therapeutic importance for their antioxidant or scavenging ROS, anti-inflammatory, and antimicrobial properties.

The properties of Ag-based nanoparticles (antimicrobial and anti-inflammatory) have undergone extensive investigation. Recent research has revealed the robust antioxidant activity of nanoparticles, which has expanded their application into the realm of antioxidant therapy. This activity is attributed to their ability for free radical scavenging and lipid peroxidation inhibition [222]. Moreover, their surface chemistry allows for easy conjugation with various antioxidants, enhancing bioactivity and stability [223].

Silver-based nanoparticles can be effectively conjugated with antioxidants, leading to enhanced stability and bioactivity, which in turn improves their therapeutic efficacy. Antioxidants like quercetin, curcumin, and resveratrol have been successfully linked with silver nanoparticles using electrostatic interaction, covalent bonding, and physical adsorption [224]. The choice of method depends on the properties of the antioxidant and the silver nanoparticles.

The antioxidant efficacy of silver-based nanoparticles arises from their capability to effectively scavenge free radicals and impede lipid peroxidation processes [225]. The silver nanoparticles can directly interact with the free radicals, neutralizing them and preventing oxidative damage. Moreover, the conjugation of antioxidants with silver nanoparticles can enhance their antioxidant activity, resulting in improved therapeutic efficacy. Several studies have reported the antioxidant activity of nanoparticles like silver and gold along with their conjugates in laboratory-based research studies [226]. It has been reported that AgNPs exhibited a substantial reduction in lipopolysaccharide (LPS)-induced oxidative damage and TNFα production in microglial cells [227]. Silver-based nanoparticles and their antioxidant conjugates have diverse potential applications. In the pharmaceutical sector, they enhance drug formulation stability and therapeutic efficacy. However, AgNPs and their antioxidant conjugates need to be studied more regarding ROS-associated neurodegeneration. The antioxidant conjugates of silver nanoparticles hold promising prospects for treating various ailments, including cancer, neurodegenerative problems, and cardiovascular disorders.

### 4.4. Platinum-Based NPs (PtNPs) and Their Antioxidant Conjugates in Oxidative Stress

Platinum (Pt) is a clinically used catalyst for chemotherapy medications like cisplatin and is used in synthetic chemistry for hydrogenation and oxidation processes. Previous research has indicated that platinum possesses the potential as a mimic of SOD and CAT in the treatment of diseases associated with free radicals [228]. Pt exhibits catalytic activity in converting superoxide (O_2_^-^) to hydrogen peroxide (H_2_O_2_) and further converting H_2_O_2_ to water (H_2_O) and molecular oxygen (O_2_). Pt-conjugated NPs showed better antioxidant activity against ROS [229]. PtNPs conjugated with citrate showed potential antioxidative activity by scavenging intracellular ROS without a cytotoxic effect. It also showed modulation in the transcription of different genes affected by LPS treatment [230]. 

At a concentration of 1%, Pt shows enhanced cellular endocytosis and exerts an antioxidant effect similar to unconjugated nano-P. This leads to increased bioavailability and reduced toxicity compared to prebinding levels. TAT-Pt NPs have the potential to improve nematode survival from enhanced endogenous ROS [231]. A PtNP-conjugated nanomotor operated based on the local concentration gradient of oxygen (O_2_) showed the breakdown of hydrogen peroxide (H_2_O_2_). The experimental findings highlighted the nanomotor’s ability to effectively counteract gastric acid while simultaneously releasing medication, leading to a favorable therapeutic outcome without any observed toxicity [232]. PtNPs exhibited excellent free radical scavenging activity and reduced oxidative injury generated by H_2_O_2_ [233]. The neuroprotective role of PtNPs and their conjugates have been observed in various animal models with neurological challenges, such as middle cerebral artery occlusion (MCAO) [234] and PD [235], but the detailed underlying mechanisms are obscured. Hence, nanomotors based on PtNPs can be further studied to reduce oxidative stress-associated neurological diseases.

### 4.5. Antagonistic Effect of Copper-Based NPs (CuNPs) and Their Antioxidant Conjugates against Oxidative Stress

Copper (Cu) exerts a multifaceted influence on cellular processes, augmenting the enterprise of superoxide dismutase (SOD) as well as other enzymatic systems [236]. Copper (Cu) enhances enzyme detoxification mechanisms, counteracts free radicals, and modulates specific signaling pathways to preserve organism balance and avoid inflammatory disorders. Potential antioxidant nanomaterials and metal–organic frameworks (MOFs) also show promise in reducing free radicals, with copper-based MOFs exhibiting peroxidase mimetic activity by decomposing H_2_O_2_ [231,237]. 

Copper (Cu), a vital trace metal for human physiology, participates in numerous enzymatic systems, including tyrosinase and Cu-Zn SOD. Based on this understanding, the logical inference arises that copper-based nanomaterials possess the potential for scavenging ROS. Cu nanoparticles (NPs) demonstrate strong catalytic ability in eliminating H_2_O_2_ and O_2_ due to quantum confinement effects. However, they cannot neutralize OH simultaneously. In contrast, Cu_2_O NPs can deactivate both H_2_O_2_ and OH, displaying potent catalytic activity similar to peroxidase enzymes. Integrating Cu_2_O and Cu nanocrystals can create a synergistic effect, resulting in a composite with enhanced enzymatic catalytic capabilities and antioxidant properties. The effective separation of electron–hole pairs between Cu_2_O and Cu promotes the Cu_2_O coating on CuNPs, further enhancing ROS scavenging performance [156]. In their pursuit to combat an array of ROS-associated disorders, such as neuronal damage and inflammation, ultra-small Cu_5.4_O NPs (Cu_5.4_O USNPs) are distinguished by their exceptional biocompatibility, enzymatic free radical scavenging efficacy, as well as remarkable renal clearance attributes. Cu_5.4_O ultra-small nanoparticles (USNPs) exhibited remarkable oxidative damage-reducing potency, which may be observed in neurodegeneration. Notably, these particles represent a significant reduction of at least two orders of magnitude compared to previously reported nanomaterials employed for ROS-related disorder treatments. The Cu_5.4_O USNPs behaved as analogs of catalase (CAT), glutathione peroxidase (GPx), and superoxide dismutase (SOD), exhibiting broad-spectrum ROS scavenging capabilities. Moreover, the ultra-small NPs possessed excellent renal clearance properties and exerted a remarkable therapeutic effect against various ROS-linked disorders without discernible evidence of adverse effects [156,231]. Hence, because of their easy bioavailability, antioxidant activity, and excellent renal clearance, such ultra-small copper-based NPs need to be explored more in detail for their antagonistic activity against ROS and oxidative stress-induced neurodegeneration.

### 4.6. Therapeutic Role of Zinc-Based NPs (ZnNPs) and Their Antioxidant Conjugates

H_2_O_2_ contributes to cellular damage by reducing the activity of SOD in O_2_-generating systems [238]. Free radical generation and inflammation are known to cause high-level tissue-destruction mechanisms [239,240,241]. Therefore, a reduction in free radicals is crucial for curing various diseases. Zinc oxide and its conjugated NPs, a known metal oxide commonly used in coatings, paints, and cosmetic products such as sunscreens, is currently valued for its potential antioxidant, anti-inflammatory, antibacterial, and UV-protection properties [241,242,243,244,245]. The hippocampus plays a vital role in cognitive processes such as learning and memory in both rodent and human populations [246]. Anatomical or structural alterations within the hippocampus may underlie the decline in cognitive function observed in diabetic rats, potentially attributed to hyperglycemia-induced changes [247]. Administration of CurNP and zinc oxide nanoparticles (ZnNPs) to the animal model with hippocampal modifications showed significant improvements in spatial memory performance. In the rat model of type 2 diabetes mellitus (T2DM), administration of CurNP and ZnNP resulted in increased time spent in the target quadrant and a twofold increase in crossing the platform zone. These nanoparticles can potentially safeguard cognitive functions related to spatial and memory abilities in the diabetic rat model [246]. In AD, oxidative damage is an early indication and a result of amyloid-beta (Aβ) accumulation in the brain. ZnNPs demonstrated significant efficacy in diabetic rats by restoring the activity of GPx, SOD, and catalase (CAT). They also increased reduced glutathione (GSH) production while reducing levels of free radicals [246]. Recently, ZnNPs synthesized using the rhizhome extract of *Zingiber officinale* and aqueous extract of *Andrographis alata* exhibited a potential anti-Alzheimer activity by inhibiting Acetylcholinesterase (AChE) and Butyrylcholinesterase (BChE) as well as antioxidative action in vitro [248,249]. Various drug (docosahexaenoic acid and osthol)-loaded ZnNPs showed promising neuroprotective effects by modulating synaptic proteins such as postsynaptic density-95 (PSD-95); synapsin-1; synaptophysin (SYP); and brain neurotransmitters such as noradrenaline, serotonin, and dopamine [250,251]. Further detailed studies are needed to develop potential ZnO-conjugated NPs for treating neurological diseases.

### 4.7. Aluminum-Based NPs (AlNPs) and Their Antioxidant Conjugates against Oxidative Stress

In recent years, aluminum-based nanoparticles (AlNPs) have garnered considerable interest owing to their distinctive characteristics, encompassing their substantial surface area, elevated reactivity, and biocompatibility. These inherent properties position AlNPs as highly promising contenders for a diverse array of applications, encompassing biomedical, environmental, and energy sectors. However, their reactive nature also makes them prone to oxidation, leading to reduced stability and potential toxicity. To overcome these challenges, researchers have fabricated air-sensitive aluminum NPs, resulting in enhanced stability and improved reduction potential [252]. 

Antioxidant conjugation of AlNPs can improve their stability and enhance their therapeutic efficacy. Various Al-based NPs exhibited potential antioxidant activity, such as grapefruit peel-fabricated Al_2_O_3_ NPs [253], Irganox 1010-fabricated Al_2_O_3_–poly(ethylene-co-butyl acrylate) nanocomposites [254], and Al_2_O_3_ NPs synthesized via the laser ablation technique [255]. The conjugation of antioxidants with NPs or entrapped in nanogel can also enhance their bioavailability and facilitate their cellular uptake, in addition to diminishing free radicals [256]. Dual-epitope synthetic long peptides (SLPs)-loaded AlNPs [257] showed potential application in the biomedical field (anti-tumor immunotherapy). However, very few studies showed the potential therapeutic activity of Al-conjugated NPs. Hence, there is a need to study the antioxidant activity and free radical scavenging activity of AlNPs to protect neurons and the brain from oxidative injury.

### 4.8. Beneficial Action of Selenium-Based NPs (SeNPs) and Their Antioxidant Conjugates in Oxidative Stress

Selenium refers to a fundamental known element that exerts a pivotal responsibility for the preservation of men’s health and homeostasis. However, excessive intake of selenium can lead to toxic effects. To address this issue, selenium nanoparticles (SeNPs) have been extensively studied as a potential alternative to selenium supplements due to their superior bioavailability, safety, and efficacy [258]. The dietary requirement of selenium is about 55–90 µg/day [259], whereas the maximum tolerable limit is about 1000–1500 µg/day, which is in contrast with the human tolerable limit of 400–500 µg/day [260]. However, this limit depends upon various factors such as biological variation, age, gender, nutritional status, and health condition [260,261]. SeNPs have been shown to possess potent antioxidant activity, making them promising candidates for antioxidant therapy [262]. In the cerebral cortex containing neurons and astrocytes, SeNPs inhibited oxygen and glucose deprivation-induced (ischemia-like conditions) necrosis and apoptosis at doses of 0.5 and 2.5 µg/mL [263]. Treatment of SeNPs (20 nm) at doses of 0.05, 0.5, or 4 mg/kg body weight (bw)/day for 28 days showed no effects on brain neurotransmitters, liver histopathology, and hematological indices [264]. Treatment of SeNPs (0.5 mg/kg) for 2 months ameliorated deltamethrin-induced alterations in the brain redox state, brain histopathology, and reduced brain acetylcholinesterase (AChE) levels in rats [265]. This represents the potential neuroprotective role of SeNPs. Conjugating antioxidants with SeNPs enhances stability and bioactivity, leading to improved therapeutic efficacy. Various antioxidants, like quercetin, curcumin, and resveratrol, have been successfully conjugated with SeNPs [266]. The antioxidant potential of SeNPs can be ascribed to their remarkable capacity to effectively scavenge free radicals and impede lipid peroxidation. It has been documented that a deficiency of selenium leads to neurological diseases such as AD, PD, seizures, and epilepsy [267,268]. It has also been documented that SeNPs not only easily cross the BBB but also prevent Aβ aggregation [269,270]. Chlorogenic acid (CGA)-conjugated SeNPs (CGA-SeNPs) showed promising oxidative injury-reducing activity by scavenging ROS and antiaggregatory properties by inhibiting Aβ40 aggregation. These NPs (60 μg/mL) also exhibited protective action against Aβ aggregation-induced PC12 cell death, representing strong neuroprotective action [271].

The SeNPs can directly interact with the free radicals, neutralizing them and preventing oxidative damage [272]. Moreover, the conjugation of antioxidants with SeNPs can enhance their antioxidant activity, improving therapeutic efficacy. Several studies have reported the antioxidant activity of SeNPs and their conjugates in laboratory-based research studies [262]. The potential applications of SeNPs and their antioxidant conjugates exhibit an extensive and diverse range. These nanoparticles find utility within the food industry for preventing lipid oxidation, extending the shelf life, and enhancing food quality [273]. SeNPs can serve as antioxidants in pharmaceutical drug formulations, improving stability and efficacy. Their antioxidant properties make them suitable for treating various ailments, including cancer, neurodegenerative disorders, and cardiovascular diseases [274]. Several studies have documented strong evidence that SeNPs and their conjugates with different molecules (resveratrol [275], epigallocatechin-3-gallate [276], curcumin [277], penicillamine [278], chlorogenic acid [271], morin [279], and sialic acid [280]) showed a significant reduction in free radicals or ROS, Aβ aggregation, as well as disaggregation of preformed Aβ fibrils into harmless oligomers. Due to the antioxidant and Aβ disaggregation activity of SeNPs, they can be employed in treating various neurological disorders.

### 4.9. Use of Manganese-Based NPs (MnNPs) and Their Antioxidant Conjugates to Reduce Oxidative Stress

Mn_3_O_4_ nanozymes or manganese dioxide (MnO_2_) nanoparticles have antioxidant enzyme-like capabilities [281,282]. When compared to CeO_2_, which is the antioxidant utilized most often, Mn_3_O_4_ nanozymes were found to be more efficient at removing ROS. Besides ROS removal efficacy in vitro, MnNPs showed a strong protective effect in live mice against ROS-induced inflammation in the ear [283]. Mn_3_O_4_ nanozymes exhibited multienzyme mimetic activity to reduce free radicals and represent promising therapeutic nanomedicine for the treatment of ROS-related diseases [284]. The exceptional multienzyme activity of Mn_3_O_4_ nanozymes can be attributed to factors such as mixed valence states of Mn^2+^/Mn^3+^, oxidation tolerance, large surface area, and extraordinarily large pore size. These characteristics collectively contribute to the efficient catalytic performance of Mn_3_O_4_ nanozymes [285]. Treatment of an AD represented-mouse model with MnNPs showed a noteworthy reduction in oxidative imbalance, neuroinflammation, and amyloid β plaques in the brain. An MRI study also revealed microvessel integrity with clearance of amyloid β [286]. In vivo (allodynia in Wistar rats via partial sciatic nerve transection) and in vitro in bone marrow-derived macrophage cells studies revealed that treatment of MnNPs significantly reduces oxidative injury, allodynia, and expression of inflammatory pain mediator cyclooxygenase-2 [287]. Such ameliorative changes promise that the best-suited treatment for neurological diseases associated with oxidative stress involves using MnNPs.

### 4.10. Potential Role of Titanium-Based NPs (TiNPs) and Their Antioxidant Conjugates in Oxidative Stress

The unique physicochemical characteristics of titanium-based nanoparticles (TiNPs), including their substantial surface area-to-volume ratio, catalytic activity, and biocompatibility, have rendered them versatile and prevalent across diverse domains. Their extensive applications encompass biomedicine, cosmetics, food packaging, and environmental remediation. The conjugation of polyphenols with TiNPs can improve their biocompatibility, increase their stability in biological environments, and reduce their toxicity by scavenging free radicals. The incorporation of polyphenol-functionalized TiNPs holds promise for augmenting their therapeutic effectiveness in a range of ailments, such as cancer, cardiovascular diseases, and neurodegenerative disorders [288]. TiNPs enhanced antioxidant metabolism activity with ease and safe vesicular internalization by the cells. These particles also suppressed the expression of proteins involved in apoptosis, such as Casp8, NF-κB, MAPK14, and JUN [289]. Various research studies have revealed the capability of polyphenol-functionalized TiNPs or polymeric NPs as effective antioxidant agents. Titanium dioxide NPs synthesized from fruit peel exhibited dose-dependent antioxidant activity via scavenging free radicals in vitro [290]. Marigold is an antioxidant known to reduce ROS. Marigold-functionalized TiNPs showed strong free radical scavenging activity with no side effects [291]. Polyphenol-functionalized polymeric NPs or TiNPs have shown great potential as effective antioxidant agents and in the medicaments of numerous disorders such as cancer and cardiovascular disease [292,293]. 

Biosynthesized TiO_2_ nanoparticles from the *Citrus aurantium* fruit peel extract showed promising anti-apoptotic effects in mammalian neuronal cell lines [294]. Polyphenol conjugation with polymeric nanoparticles improves biocompatibility, increases stability, and reduces toxicity by scavenging free radicals. Further research is needed to fully understand the therapeutic potential and underlying mechanisms of action of polyphenol-functionalized TiNPs in oxidative stress-associated neurodegenerative diseases.

### 4.11. Iron-Based NPs (IONPs) and Their Antioxidant Conjugates to Abolish Oxidative Stress

Iron oxide (Fe_3_O_4_) NPs are highly biocompatible and hence have been widely used in drug delivery [295,296], therapeutic [297,298], bioimaging [296,299], as well as diagnostic purposes [298,300]. The antioxidant properties of IONPs were studied in two cell lines (L929 cells and PC12), and they were found to be in the cytoplasm. IONPs exhibited catalase-like characteristics that may effectively eliminate extra ROS within cells, preventing them from oxidative damage and H_2_O_2_-induced death. On PC12 cells, Fe_3_O_4_ nanoparticles’ neuroprotective abilities were evaluated in vitro. PC12 cells, derived from the neural crest and comprising neuroblastic and eosinophilic cells, upon stimulation with nerve growth factor (NGF), can undergo differentiation resembling neurons [301]. Differentiated PC12 cells are commonly used in neuroprotective assays related to PD [302]. The compound 1-methyl-4-phenylpyridinium ion (MPP+) induces oxidative damage and cell death. In cellular studies, IONPs have demonstrated the ability to prevent MPP+-induced cell death and reduce the activation of caspase-3 and α-synuclein, two proteins associated with cell death in PD. The elav-Gal4 and UAS-A lines were crossed to achieve neuronal-specific production of the A peptide in the Drosophila AD model, allowing for targeted expression of A within the nervous system. Fruit flies from the AD model had a greater capacity for climbing and lived longer than untreated ones when fed food with IONPs. The strong antioxidant qualities of IONPs in postponing animal aging and reducing ROS-induced neurotoxicity were further confirmed by the Drosophila AD model [301]. Fe_3_O_4_ nanozymes showed a potent mitigative effect against d-galactose-induced oxidative damage of the neuroblast as well as apoptosis and autophagy in the hippocampal dentate gyrus [303]. IONPs showed potential application in the diagnosis and treatment of different neurodegenerative diseases, such as AD, PD, and ALS [304]. Quercetin-loaded NPs synthesized from an iron oxide core with the coat of β-cyclodextrin and pluronic F68 polymer showed a significant decline in neuronal loss and seizure in a model exhibiting epilepsy disorder (pentylenetetrazole-induced kindling model) [305]. Based on all the above studies, it is very clear that the iron-based NPs and Fe_3_O_4_ nanozymes have the potential to protect neurons from oxidative injury and ROS; however, more comprehensive studies are also required to further elucidate their role.

### 4.12. Ameliorative Action of Cerium-Based NPs (CeNPs) and Their Antioxidant Activity against ROS-Mediated Oxidative Stress

Cerium oxide nanoparticles (CeNPs) and ultrasmall CeNPs exhibit potent antioxidant properties, and they have a strong capability to treat diseases developed due to life-threatening ROS [306]. CeNPs, also known as nanoceria or ceria NPs, have a large therapeutic potential. Their ability to resemble biological antioxidants is ascribed to Ce^3+^ ions in CeO_2_. Ce^4+^ and Ce^3+^, which are less stable in their oxide forms, coexist and form a redox couple that gives the material its catalytic activity. There are enough oxygen vacancies to make up for Ce^3+^’s lower positive charge. On the surface of ceria, there are more oxygen vacancies and Ce^3+^ ions than there are in the bulk of the material. Consequently, Ce^3+^ ions increase the density, followed by enhancing redox potential activation. Through the reversible binding of oxygen along with the conversion of Ce^3+^ to Ce^4+^ on their surface, Ceria NPs neutralize free radicals. As a result of their SOD- and CAT-biomimetic properties, ceria NPs defend cells from the most prevalent ROS, including O_2_, OH, and H_2_O_2_ [307,308,309]. 

Previous research has shown that monocytes may internalize cerium NPs, which have surface oxygen vacancies and act as scavengers for free radicals [231,310]. Nanoceria can influence intracellular ROS levels in this way, especially when the monocytes are stimulated [274]. This stops one of its main sources from releasing too much ROS into the environment. When constructed on a TiO_2_ substrate and considering Ceria, nanoparticles with fullerene-like structures have the powerful ability to scavenge free radicals (ROS) and reduce oxidative imbalance [311]. The significance of water-soluble cerium nanoparticles (NPs) topically promotes the complete healing of deep wounds in mouse skin by stimulating the regulation and modulation of fibroblasts, movement of vascular endothelial cells, and migration of keratinocytes. These processes involve the removal of intracellular reactive oxygen species (ROS) and the inhibition of apoptosis pathways activated by hydrogen peroxide (H_2_O_2_) [312]. By reducing ROS-induced cell death, the cellular uptake of nanoceria plays a significant role in promoting wound healing. Nanoceria treatments help the skin to close and revascularize, according to a mouse wound-healing study [231]. The presence of a large quantity of ROS in the neurons and cerebrovascular cells is a deteriorating factor in the progression of neurological disorders such as AD. It has been reported that the treatment of human cerebral microvascular endothelial cells (hCMEC/D3), exposed to amyloid-β peptide (Aβ) oligomers with CeNPs exhibited restoration of the enhanced levels of ROS and represented potent antioxidant activity [313]. Exposure of neuronal cells to ischemia-like environments enhanced the mitochondria damage-based ROS production and altered calcium homeostasis. These deteriorating conditions were ameliorated by cerium-based nanomaterials with lipid self-assembling nanoparticles and represented an excellent antioxidant agent to treat various neurodegenerative diseases and associated life-threatening conditions due to free radicals [314]. Above all, the studies represented the potent antioxidative and oxidative stress-reducing activity of cerium-based NPs; however, more detailed in vivo studies are required to explore their neuroprotective activity. Table 3 represents the role of various NPs and their conjugates in neuroinflammation and neurodegeneration-associated pathologies.

**Figure 2:**
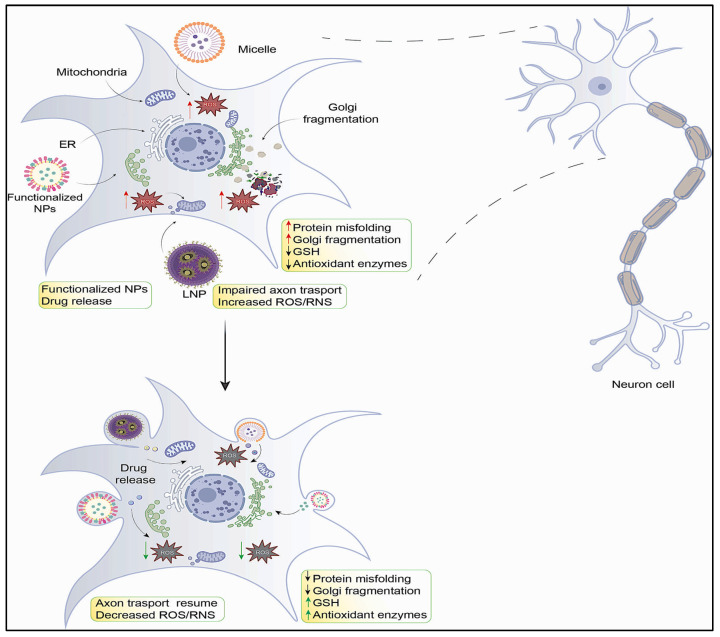
Mechanisms of action of different functionalized NPs. Schematic representation of internalization of different NPs or specific antioxidants in damaged neurons and their action on various cellular organelles to reduce ROS or scavenge ROS.

Figure 2 represents the internalization of various functionalized NPs and career molecules for the delivery of antioxidants or drugs to scavenge ROS and reduce oxidative stress, which in turn protects neurons from oxidative damage. 

## 5. Nanotheranostics for Oxidative Stress

Nanotheranostic molecules represent key opportunities in the diagnostic and therapeutic challenges faced in various neurodegenerative diseases due to oxidative stress and other factors. Nanoparticle-associated delivery of various drugs has been considered an assuring novel opportunity to treat neurodegenerative diseases. For this reason, various drug delivery systems and nanoparticles (lipid-based nanomaterials, magnetic nanoparticles, oxide/metallic nanoparticles, aptamer, gold/silver nanoparticles, and dendrimer-based nanoparticles) can be functionalized to achieve regulated, coordinated, and long-term release of medicine or drug to the precise organ or tissue or cells [332,333]. Nanotheranostics involve the administration of nanotheranostic molecules consisting of the outer shield and covering the medicine or drug in the core. Such nanotheranostic molecules can disintegrate after approaching the specific target area of the body and subsequently release the medicine or drug [334,335,336,337]. Such nanotheranostic molecules can be custom-made based on specific requirements, such as the type of stimuli and tissue or cell-specific microenvironment of the disease, to provide personalized therapeutics. Stimuli can be oxidative injury or alteration of pH and temperature of the microenvironment, etc. 

It has been reported that oxidative imbalance decreases the pH by affecting the exchange of ions across the membrane in neurons [338]. The normal pH of the extracellular environment in tissue and blood is approximately 7.4, maintained by regular biochemical cycles like glycolysis, the citric acid cycle, and the electron transport chain. Elevated levels of free radicals and reduced pH due to lipopolysaccharide have been reported to trigger the release of encapsulated curcumin from curcumin-loaded nanoparticles. The released curcumin exhibited potent anti-inflammatory and antioxidant activity, reducing ROS in an ankle-inflamed rodent model [339]. poly(ethyleneglycol)-block-poly(2-(diisopropylamino)ethyl methacrylate) (PEG-b-PDPA–a pH-responsive diblock copolymer) and D-α-tocopherylpolyethyleneglycol-1000-succinate (TPGS—a vitamin E derivate)-derived micelles proved the best chemotherapeutic agent against doxorubicin-induced cytotoxicity by targeting the transmembrane potential of mitochondria, which can generate oxidative imbalance [340]. Most of the pH-responsive nano-theranostics and/or nanostructures have been applied to different diseases [338,341,342,343,344]. Such pH-responsive polymers are associated with ionizable moieties or possess acid–labile linkages. Ionizable moieties-associated polymers are prepared by the incorporation of acidic groups or basic functional groups. Alteration of pH in the biological environment can either protonate or deprotonate such ionizable moieties [345,346] and release the drug of interest (DOI). 

**Figure 3:**
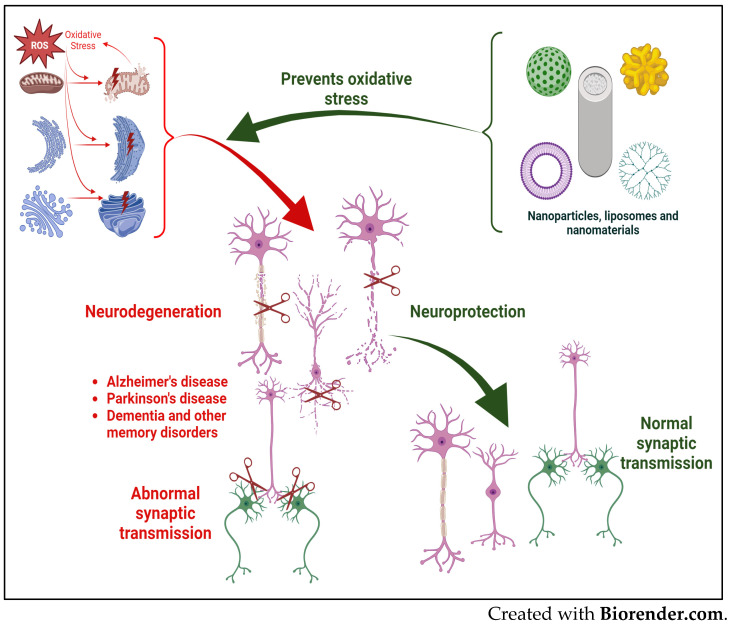
Mechanisms of neuronal damage and its protection by various NPs. Various NPs and their functionalization can protect neuronal cells from neurodegeneration at the structural or functional level by abrogating ROS.

ROS-responsive nanoparticles support the discharge or release of effective drugs and protect against oxidative damage. Thus, they prevent groups of cells, tissue, or organs from oxidative damage. ROS-responsive nanoparticles have been formulated using various materials such as triphenylphosphine [347], graphene oxide [348,349,350], thioketal [351,352,353,354,355], selenium [356], and tellurium [357]. Glutathione (GSH) is a crucial non-enzymatic antioxidant present in cells and tissues. It is increased during oxidative stress and certain pathological conditions. GSH/ROS responsive nanoparticles showed promising release of the key drug present in the core via thiolysis or hydrolysis in the presence of GSH or ROS-oxidation of the linker, respectively [358,359,360]. ROS-responsive thioketal nanoparticles (TKNs) made from poly-(1,4-phenyleneacetone dimethylene thioketal) were synthesized for delivering siRNA against proinflammatory cytokines to regions with elevated ROS levels. Oral administration of these TKNs demonstrated protection against ulcerative colitis [361]. The singlet oxygen-responsive crosslinker has been associated with hyperbranched polyphosphate for the successful release of the drug of interest with great biocompatibility [362]. A ROS-activated and mitochondria-targeting drug delivery system using ZnPc/CPT-TPPNPs was developed with a ROS-sensitive thioketal linker associated with camptothecin as well as triphenylphosphonium, a positively charged compound with a membrane-penetrating property [363]. Hydrogen peroxide (H_2_O_2_) (an active ROS)-responsive CO_2_ bubble-producing poly(vanillin oxalate) nanoparticles showed antioxidant, anti-inflammatory, and anti-apoptotic activity. These nanoparticles possess a vanillin molecule that has anti-inflammatory activity, showing the best nanotheranostic activity against ROS [364]. Figure 3 represents the mechanism of neuronal damage and their protection by various NPs.

The development of ROS-responsive nanoparticles and their therapeutic activity needs to be explored more by using different in vitro and in vivo models for various ROS-dependent pathological conditions.

## 6. Conclusions and Future Prospectus

The valuable features of nanoparticles and/or nanomaterials offer innovative diagnostic tools in addition to their therapeutic potential. In the past decade, numerous antioxidant-conjugated NPs and oxidative stress-responsive NPs have been developed as nanotheranostics. They have also been improved continuously with no side effects and high efficacy as well as efficiency via the addition of various reducing functional groups. Oxidative injury and ROS-responsive drug delivery systems effectively shelter the active drugs or medicines in the core and deliver them at the site of action. Such systems protect drugs from quick decomposition and prolong their bioavailability in the body. Furthermore, such antioxidant-conjugated NPs and nano-delivery systems demonstrate the potential nanotheranostics and exhibit their therapeutic applications to treat various oxidative damage-associated diseased conditions, such as neuroinflammation, neurodegeneration, AD, PD, and ischemia/reperfusion-induced brain damage. However, the early, delayed, or incomplete release of effective therapeutic agents may also adversely affect their therapeutic efficacy. Hence, further detailed preclinical (in vitro and in vivo) and clinical studies of such antioxidant-conjugated NPs and nanotheranostic-based nanomedicine are indispensable. 

## Figures and Tables

**Table 2:** Impact of nanoparticle size on biodistribution and pharmacokinetics.

Vector	Proposed Study	Results	References
AuNPs	In vivo biodistribution studies	10 nm-sized AuNPs were discovered to localize in diverse organs.	[173]
PEG-PHDCA nanoparticles	Particle size effects on the rHuTNF-a loaded PEGPHDCA nanoparticles’ elimination half-lives	Nanoparticles with the longest circulation times are 80 nm in size.	[174]
AuNPs coated with thioctic acid terminated PEG	In vivo biodistribution studies	The 20 nm AuNPs were detected in naked mice with subcutaneous A431 squamous malignancies.	[175]
PEGylated nanoparticles	Analysis on how size affects pharmacokinetics and biodistribution	The PLA-PEG composition >70 nm exhibited prolonged systemic circulation and reduced liver uptake, whereas that of <70 nm exhibited accumulation in the liver.	[176]
PEG-modified poly-e-caprolactone (PCL) nanoparticles	Tamoxifen delivered systemically using PCL nanoparticles for breast cancer patients	PEG modification improved circulation time and decreased particle size and agglomeration.	[177]
Block copolymer micelles (BCMs)	In vivo study on size distribution	Compared to BCMs of 60 nm, BCMs of 25 nm were promptly removed from the plasma.	[178]
RhB-CMCNP and RhB-CHNP	In vivo biodistribution studies	In tumor cells, negative charges and particles smaller than 150 nm tended to accumulate more quickly.	[179]

Abbreviations: RhB-CMCNP—Rhodamine B carboxymethyl chitosan grafted nanoparticles; RhB-CHNP—Rhodamine B chitosan hydrochloride grafted nanoparticles.

**Table 3:** Role of various NPs and their conjugates in neuroinflammation and neurodegeneration-associated pathologies.

Nanoparticles	Loaded or Conjugated Molecule	Disease Model	Mechanism of Action	References
AuNPs	-	AD	Inhibits amyloid beta (Aβ) peptide aggregation	[212]
AuNPs	-	AD	Prevents spatial memory, oxidative stress in brain, neuroinflammation, restores antioxidant status (SOD, catalase activities, and GSH levels) in brain	[209]
AuNPs	Anthocyanin	AD	Reduces Aβ-induced neuroinflammatory and neuroapoptotic markers	[315]
AuNPs	Root extract of *Paeonia moutan*	PD	Alleviates neuroinflammation and restores motor coordination	[194]
AuNPs	FM19G11 (a hypoxia-inducible factor modulator)	ALS	Enhances proliferation and self-renewal of ependymal Stem Progenitor Cells (epSPCs)	[316]
AgNPs	Aqueous extract of *N. khasiana* leaf	AD	Prevents spatial memory and recognition deficit	[317]
AgNPs	-	PD	Reduces ROS and neuroinflammation	[227]
PtNPs	-	Brain damage due to ischemic stroke	Prevents ROS production, brain damage, and neurological function	[234]
PtNPs	Extract of *Bacopa monnieri* leaf	PD	Enhances GSH levels, activities of antioxidant enzymes, and locomotor activity	[235]
CuNPs	Phenylalanine (Phe)	PD	Eliminates ROS and rescues memory loss	[318]
CuNPs	-	AD	Reduces Aβ burden, oxidative damage, and memory deficit	[319]
Zinc-Polymeric NPs	-	AD	Reduces plaque size and normalizes hyperlocomotion	[320]
ZnNPs	Myco-fabricated	AD	Improves learning and memory; increases antioxidant enzyme activity	[321]
SeNPs	Resveratrol	AD	Increases various neuroprotective activities, such as antioxidants, anti-neuroinflammation, and neurocognition	[322,323]
SeNPs	Peptides, curcumin, Resveratrol	AD	Suppresses Aβ aggregation and ROS generation	[270,277,323]
SeNPs	Ascorbic acid and chitosan	PD	Enhances antioxidant activity and motor function	[324]
SeNPs	Rutin	Epilepsy	Inhibits neuronal apoptosis and enhances antioxidant defense	[325]
MnNPs	-	AD	Reduces hypoxia, neuroinflammation, Aβ plaques, and oxidative damage	[286]
IONPs	Quercetin	AD	Improves learning and memory and inhibits progression of cognitive impairment	[326]
IONPs	Hyaluronic acid nanogels	AD	Impedes Aβ aggregation and induces disaggregation of Aβ fibrils	[327]
IONPs	-	PD	Improves gait, postural stability, and mitochondrial function	[328]
CeNPs	Triphenylphosphonium	AD	Improves mitochondrial function and suppresses neuronal death	[329]
CeNPs	-	AD	Decreases human Tau gene expression and restores superoxide dismutase activity	[330]
CeNPs	-	Traumatic Brain Injury	Preserves antioxidant systems, reduces free radical damage, and improves cognition	[331]

## Data Availability

Not applicable.

## References

[1] Betteridge D.J. (2000). What is oxidative stress?. Metabolism.

[2] Lin K.T., Xue J.Y., Sun F.F., Wong P.Y. (1997). Reactive oxygen species participate in peroxynitrite-induced apoptosis in HL-60 cells. Biochem. Biophys. Res. Commun..

[3] Lassmann H., van Horssen J. (2016). Oxidative stress and its impact on neurons and glia in multiple sclerosis lesions. Biochim. Biophys. Acta.

[4] Pacher P., Beckman J.S., Liaudet L. (2007). Nitric oxide and peroxynitrite in health and disease. Physiol. Rev..

[5] Hameister R., Kaur C., Dheen S.T., Lohmann C.H., Singh G. (2020). Reactive oxygen/nitrogen species (ROS/RNS) and oxidative stress in arthroplasty. J. Biomed. Mater. Res. B Appl. Biomater..

[6] Chen X., Guo C., Kong J. (2012). Oxidative stress in neurodegenerative diseases. Neural. Regen. Res..

[7] Fulda S., Gorman A.M., Hori O., Samali A. (2010). Cellular stress responses: Cell survival and cell death. Int. J. Cell. Biol..

[8] Obeso J.A., Rodriguez-Oroz M.C., Benitez-Temino B., Blesa F.J., Guridi J., Marin C., Rodriguez M. (2008). Functional organization of the basal ganglia: Therapeutic implications for Parkinson’s disease. Mov. Disord..

[9] Gandhi S., Abramov A.Y. (2012). Mechanism of oxidative stress in neurodegeneration. Oxid. Med. Cell. Longev..

[10] Li J., O W., Li W., Jiang Z.G., Ghanbari H.A. (2013). Oxidative stress and neurodegenerative disorders. Int. J. Mol. Sci..

[11] Xiang W., Schlachetzki J.C., Helling S., Bussmann J.C., Berlinghof M., Schaffer T.E., Marcus K., Winkler J., Klucken J., Becker C.M. (2013). Oxidative stress-induced posttranslational modifications of alpha-synuclein: Specific modification of alpha-synuclein by 4-hydroxy-2-nonenal increases dopaminergic toxicity. Mol. Cell. Neurosci..

[12] Guo J.D., Zhao X., Li Y., Li G.R., Liu X.L. (2018). Damage to dopaminergic neurons by oxidative stress in Parkinson’s disease (Review). Int. J. Mol. Med..

[13] Ma T., Klann E. (2012). Amyloid beta: Linking synaptic plasticity failure to memory disruption in Alzheimer’s disease. J. Neurochem..

[14] Parajuli B., Sonobe Y., Horiuchi H., Takeuchi H., Mizuno T., Suzumura A. (2013). Oligomeric amyloid beta induces IL-1beta processing via production of ROS: Implication in Alzheimer’s disease. Cell Death Dis..

[15] Ermak G., Davies K.J. (2002). Calcium and oxidative stress: From cell signaling to cell death. Mol. Immunol..

[16] Shichiri M. (2014). The role of lipid peroxidation in neurological disorders. J. Clin. Biochem. Nutr..

[17] Devine M.J., Kittler J.T. (2018). Mitochondria at the neuronal presynapse in health and disease. Nat. Rev. Neurosci..

[18] Hollensworth S.B., Shen C., Sim J.E., Spitz D.R., Wilson G.L., LeDoux S.P. (2000). Glial cell type-specific responses to menadione-induced oxidative stress. Free Radic. Biol. Med..

[19] Douarre C., Sourbier C., Dalla Rosa I., Brata Das B., Redon C.E., Zhang H., Neckers L., Pommier Y. (2012). Mitochondrial topoisomerase I is critical for mitochondrial integrity and cellular energy metabolism. PLoS ONE.

[20] Chen J.Q., Yager J.D., Russo J. (2005). Regulation of mitochondrial respiratory chain structure and function by estrogens/estrogen receptors and potential physiological/pathophysiological implications. Biochim. Biophys. Acta.

[21] Selivanov V.A., Votyakova T.V., Pivtoraiko V.N., Zeak J., Sukhomlin T., Trucco M., Roca J., Cascante M. (2011). Reactive oxygen species production by forward and reverse electron fluxes in the mitochondrial respiratory chain. PLoS Comput. Biol..

[22] Barja G. (2013). Updating the mitochondrial free radical theory of aging: An integrated view, key aspects, and confounding concepts. Antioxid. Redox Signal..

[23] Wu Y., Chen M., Jiang J. (2019). Mitochondrial dysfunction in neurodegenerative diseases and drug targets via apoptotic signaling. Mitochondrion.

[24] Bubber P., Haroutunian V., Fisch G., Blass J.P., Gibson G.E. (2005). Mitochondrial abnormalities in Alzheimer brain: Mechanistic implications. Ann. Neurol..

[25] Branch S.Y., Chen C., Sharma R., Lechleiter J.D., Li S., Beckstead M.J. (2016). Dopaminergic Neurons Exhibit an Age-Dependent Decline in Electrophysiological Parameters in the MitoPark Mouse Model of Parkinson’s Disease. J. Neurosci..

[26] Hoglinger G.U., Lannuzel A., Khondiker M.E., Michel P.P., Duyckaerts C., Feger J., Champy P., Prigent A., Medja F., Lombes A. (2005). The mitochondrial complex I inhibitor rotenone triggers a cerebral tauopathy. J. Neurochem..

[27] Melov S., Adlard P.A., Morten K., Johnson F., Golden T.R., Hinerfeld D., Schilling B., Mavros C., Masters C.L., Volitakis I. (2007). Mitochondrial oxidative stress causes hyperphosphorylation of tau. PLoS ONE.

[28] Mandal P.K., Saharan S., Tripathi M., Murari G. (2015). Brain glutathione levels—A novel biomarker for mild cognitive impairment and Alzheimer’s disease. Biol. Psychiatry.

[29] Shukla D., Mandal P.K., Tripathi M., Vishwakarma G., Mishra R., Sandal K. (2020). Quantitation of in vivo brain glutathione conformers in cingulate cortex among age-matched control, MCI, and AD patients using MEGA-PRESS. Hum. Brain Mapp..

[30] Cunha-Oliveira T., Montezinho L., Mendes C., Firuzi O., Saso L., Oliveira P.J., Silva F.S.G. (2020). Oxidative Stress in Amyotrophic Lateral Sclerosis: Pathophysiology and Opportunities for Pharmacological Intervention. Oxid. Med. Cell. Longev..

[31] Martin L.J. (2010). The mitochondrial permeability transition pore: A molecular target for amyotrophic lateral sclerosis therapy. Biochim. Biophys. Acta.

[32] Zhu X., Castellani R.J., Moreira P.I., Aliev G., Shenk J.C., Siedlak S.L., Harris P.L.R., Fujioka H., Sayre L.M., Szweda P.A. (2012). Hydroxynonenal-generated crosslinking fluorophore accumulation in Alzheimer disease reveals a dichotomy of protein turnover. Free Radic. Biol. Med..

[33] Wang W., Zhao F., Ma X., Perry G., Zhu X. (2020). Mitochondria dysfunction in the pathogenesis of Alzheimer’s disease: Recent advances. Mol. Neurodegener..

[34] Ross W.N. (2012). Understanding calcium waves and sparks in central neurons. Nat. Rev. Neurosci..

[35] Ryan K.C., Ashkavand Z., Norman K.R. (2020). The Role of Mitochondrial Calcium Homeostasis in Alzheimer’s and Related Diseases. Int. J. Mol. Sci..

[36] Baev A.Y., Vinokurov A.Y., Novikova I.N., Dremin V.V., Potapova E.V., Abramov A.Y. (2022). Interaction of Mitochondrial Calcium and ROS in Neurodegeneration. Cells.

[37] Wang Z.T., Lu M.H., Zhang Y., Ji W.L., Lei L., Wang W., Fang L.P., Wang L.W., Yu F., Wang J. (2019). Disrupted-in-schizophrenia-1 protects synaptic plasticity in a transgenic mouse model of Alzheimer’s disease as a mitophagy receptor. Aging Cell.

[38] Montagne A., Nation D.A., Sagare A.P., Barisano G., Sweeney M.D., Chakhoyan A., Pachicano M., Joe E., Nelson A.R., D’Orazio L.M. (2020). APOE4 leads to blood-brain barrier dysfunction predicting cognitive decline. Nature.

[39] Pires M., Rego A.C. (2023). Apoe4 and Alzheimer’s Disease Pathogenesis-Mitochondrial Deregulation and Targeted Therapeutic Strategies. Int. J. Mol. Sci..

[40] di Penta A., Moreno B., Reix S., Fernandez-Diez B., Villanueva M., Errea O., Escala N., Vandenbroeck K., Comella J.X., Villoslada P. (2013). Oxidative stress and proinflammatory cytokines contribute to demyelination and axonal damage in a cerebellar culture model of neuroinflammation. PLoS ONE.

[41] Wang W.Y., Tan M.S., Yu J.T., Tan L. (2015). Role of pro-inflammatory cytokines released from microglia in Alzheimer’s disease. Ann. Transl. Med..

[42] Bahn G., Park J.S., Yun U.J., Lee Y.J., Choi Y., Park J.S., Baek S.H., Choi B.Y., Cho Y.S., Kim H.K. (2019). NRF2/ARE pathway negatively regulates BACE1 expression and ameliorates cognitive deficits in mouse Alzheimer’s models. Proc. Natl. Acad. Sci. USA.

[43] Dodson M.W., Guo M. (2007). Pink1, Parkin, DJ-1 and mitochondrial dysfunction in Parkinson’s disease. Curr. Opin. Neurobiol..

[44] Orth M., Tabrizi S.J., Tomlinson C., Messmer K., Korlipara L.V., Schapira A.H., Cooper J.M. (2004). G209A mutant alpha synuclein expression specifically enhances dopamine induced oxidative damage. Neurochem. Int..

[45] Chinta S.J., Kumar M.J., Hsu M., Rajagopalan S., Kaur D., Rane A., Nicholls D.G., Choi J., Andersen J.K. (2007). Inducible alterations of glutathione levels in adult dopaminergic midbrain neurons result in nigrostriatal degeneration. J. Neurosci..

[46] Hastings T.G. (2009). The role of dopamine oxidation in mitochondrial dysfunction: Implications for Parkinson’s disease. J. Bioenerg. Biomembr..

[47] Subramaniam S.R., Chesselet M.F. (2013). Mitochondrial dysfunction and oxidative stress in Parkinson’s disease. Prog. Neurobiol..

[48] Masato A., Plotegher N., Boassa D., Bubacco L. (2019). Impaired dopamine metabolism in Parkinson’s disease pathogenesis. Mol. Neurodegener..

[49] Heeman B., Van den Haute C., Aelvoet S.A., Valsecchi F., Rodenburg R.J., Reumers V., Debyser Z., Callewaert G., Koopman W.J., Willems P.H. (2011). Depletion of PINK1 affects mitochondrial metabolism, calcium homeostasis and energy maintenance. J. Cell Sci..

[50] Zondler L., Miller-Fleming L., Repici M., Goncalves S., Tenreiro S., Rosado-Ramos R., Betzer C., Straatman K.R., Jensen P.H., Giorgini F. (2014). DJ-1 interactions with alpha-synuclein attenuate aggregation and cellular toxicity in models of Parkinson’s disease. Cell Death Dis..

[51] Lopert P., Patel M. (2014). Brain mitochondria from DJ-1 knockout mice show increased respiration-dependent hydrogen peroxide consumption. Redox Biol..

[52] Ramesh G., MacLean A.G., Philipp M.T. (2013). Cytokines and chemokines at the crossroads of neuroinflammation, neurodegeneration, and neuropathic pain. Mediat. Inflamm..

[53] Gordon R., Albornoz E.A., Christie D.C., Langley M.R., Kumar V., Mantovani S., Robertson A.A.B., Butler M.S., Rowe D.B., O’Neill L.A. (2018). Inflammasome inhibition prevents alpha-synuclein pathology and dopaminergic neurodegeneration in mice. Sci. Transl. Med..

[54] Lee E., Hwang I., Park S., Hong S., Hwang B., Cho Y., Son J., Yu J.W. (2019). MPTP-driven NLRP3 inflammasome activation in microglia plays a central role in dopaminergic neurodegeneration. Cell Death Differ..

[55] Scudamore O., Ciossek T. (2018). Increased Oxidative Stress Exacerbates alpha-Synuclein Aggregation In Vivo. J. Neuropathol. Exp. Neurol..

[56] Wang X., Chi J., Huang D., Ding L., Zhao X., Jiang L., Yu Y., Gao F. (2020). alpha-synuclein promotes progression of Parkinson’s disease by upregulating autophagy signaling pathway to activate NLRP3 inflammasome. Exp. Ther. Med..

[57] Moujalled D., Strasser A., Liddell J.R. (2021). Molecular mechanisms of cell death in neurological diseases. Cell Death Differ..

[58] Voet S., Srinivasan S., Lamkanfi M., van Loo G. (2019). Inflammasomes in neuroinflammatory and neurodegenerative diseases. EMBO Mol. Med..

[59] Schwarz D.S., Blower M.D. (2016). The endoplasmic reticulum: Structure, function and response to cellular signaling. Cell. Mol. Life Sci..

[60] Hetz C., Saxena S. (2017). ER stress and the unfolded protein response in neurodegeneration. Nat. Rev. Neurol..

[61] Balch W.E., Morimoto R.I., Dillin A., Kelly J.W. (2008). Adapting proteostasis for disease intervention. Science.

[62] Kaushik S., Cuervo A.M. (2015). Proteostasis and aging. Nat. Med..

[63] Paschen W., Frandsen A. (2001). Endoplasmic reticulum dysfunction—A common denominator for cell injury in acute and degenerative diseases of the brain?. J. Neurochem..

[64] Breckenridge D.G., Germain M., Mathai J.P., Nguyen M., Shore G.C. (2003). Regulation of apoptosis by endoplasmic reticulum pathways. Oncogene.

[65] Rao R.V., Ellerby H.M., Bredesen D.E. (2004). Coupling endoplasmic reticulum stress to the cell death program. Cell Death Differ..

[66] Hasnain S.Z., Lourie R., Das I., Chen A.C., McGuckin M.A. (2012). The interplay between endoplasmic reticulum stress and inflammation. Immunol. Cell Biol..

[67] Grootjans J., Kaser A., Kaufman R.J., Blumberg R.S. (2016). The unfolded protein response in immunity and inflammation. Nat. Rev. Immunol..

[68] Chong W.C., Shastri M.D., Eri R. (2017). Endoplasmic Reticulum Stress and Oxidative Stress: A Vicious Nexus Implicated in Bowel Disease Pathophysiology. Int. J. Mol. Sci..

[69] Mossuto M.F. (2013). Disulfide bonding in neurodegenerative misfolding diseases. Int. J. Cell Biol..

[70] McGuckin M.A., Eri R.D., Das I., Lourie R., Florin T.H. (2010). ER stress and the unfolded protein response in intestinal inflammation. Am. J. Physiol. Gastrointest. Liver Physiol..

[71] Ron D., Walter P. (2007). Signal integration in the endoplasmic reticulum unfolded protein response. Nat. Rev. Mol. Cell Biol..

[72] Back S.H., Kaufman R.J. (2012). Endoplasmic reticulum stress and type 2 diabetes. Annu. Rev. Biochem..

[73] Ghemrawi R., Khair M. (2020). Endoplasmic Reticulum Stress and Unfolded Protein Response in Neurodegenerative Diseases. Int. J. Mol. Sci..

[74] Novikoff P.M., Novikoff A.B., Quintana N., Hauw J.J. (1971). Golgi apparatus, GERL, and lysosomes of neurons in rat dorsal root ganglia, studied by thick section and thin section cytochemistry. J. Cell Biol..

[75] Rambourg A., Clermont Y. (1986). Tridimensional structure of the Golgi apparatus in type A ganglion cells of the rat. Am. J. Anat..

[76] Rao S., Kirschen G.W., Szczurkowska J., Di Antonio A., Wang J., Ge S., Shelly M. (2018). Repositioning of Somatic Golgi Apparatus Is Essential for the Dendritic Establishment of Adult-Born Hippocampal Neurons. J. Neurosci..

[77] Horton A.C., Racz B., Monson E.E., Lin A.L., Weinberg R.J., Ehlers M.D. (2005). Polarized secretory trafficking directs cargo for asymmetric dendrite growth and morphogenesis. Neuron.

[78] Horton A.C., Ehlers M.D. (2003). Dual modes of endoplasmic reticulum-to-Golgi transport in dendrites revealed by live-cell imaging. J. Neurosci..

[79] Ye B., Zhang Y., Song W., Younger S.H., Jan L.Y., Jan Y.N. (2007). Growing dendrites and axons differ in their reliance on the secretory pathway. Cell.

[80] Fan J., Hu Z., Zeng L., Lu W., Tang X., Zhang J., Li T. (2008). Golgi apparatus and neurodegenerative diseases. Int. J. Dev. Neurosci..

[81] Cutrona M.B., Beznoussenko G.V., Fusella A., Martella O., Moral P., Mironov A.A. (2013). Silencing of mammalian Sar1 isoforms reveals COPII-independent protein sorting and transport. Traffic.

[82] Sun K.H., de Pablo Y., Vincent F., Johnson E.O., Chavers A.K., Shah K. (2008). Novel genetic tools reveal Cdk5’s major role in Golgi fragmentation in Alzheimer’s disease. Mol. Biol. Cell.

[83] Joshi G., Chi Y., Huang Z., Wang Y. (2014). Abeta-induced Golgi fragmentation in Alzheimer’s disease enhances Abeta production. Proc. Natl. Acad. Sci. USA.

[84] Castro-Alvarez J.F., Uribe-Arias S.A., Mejia-Raigosa D., Cardona-Gomez G.P. (2014). Cyclin-dependent kinase 5, a node protein in diminished tauopathy: A systems biology approach. Front. Aging Neurosci..

[85] Liazoghli D., Perreault S., Micheva K.D., Desjardins M., Leclerc N. (2005). Fragmentation of the Golgi apparatus induced by the overexpression of wild-type and mutant human tau forms in neurons. Am. J. Pathol..

[86] van Dis V., Kuijpers M., Haasdijk E.D., Teuling E., Oakes S.A., Hoogenraad C.C., Jaarsma D. (2014). Golgi fragmentation precedes neuromuscular denervation and is associated with endosome abnormalities in SOD1-ALS mouse motor neurons. Acta Neuropathol. Commun..

[87] Pottorf T., Mann A., Fross S., Mansel C., Vohra B.P.S. (2018). Nicotinamide Mononucleotide Adenylyltransferase 2 maintains neuronal structural integrity through the maintenance of golgi structure. Neurochem. Int..

[88] Joshi G., Wang Y. (2015). Golgi defects enhance APP amyloidogenic processing in Alzheimer’s disease. Bioessays.

[89] Dumont M., Ho D.J., Calingasan N.Y., Xu H., Gibson G., Beal M.F. (2009). Mitochondrial dihydrolipoyl succinyltransferase deficiency accelerates amyloid pathology and memory deficit in a transgenic mouse model of amyloid deposition. Free Radic. Biol. Med..

[90] Shi Q., Xu H., Yu H., Zhang N., Ye Y., Estevez A.G., Deng H., Gibson G.E. (2011). Inactivation and reactivation of the mitochondrial alpha-ketoglutarate dehydrogenase complex. J. Biol. Chem..

[91] Lopez-Erauskin J., Galino J., Bianchi P., Fourcade S., Andreu A.L., Ferrer I., Munoz-Pinedo C., Pujol A. (2012). Oxidative stress modulates mitochondrial failure and cyclophilin D function in X-linked adrenoleukodystrophy. Brain.

[92] Qadri R., Goyal V., Behari M., Subramanian A., Datta S.K., Mukhopadhyay A.K. (2021). Alteration of Mitochondrial Function in Oxidative Stress in Parkinsonian Neurodegeneration: A Cross-Sectional Study. Ann. Indian Acad. Neurol..

[93] Reddy P.H. (2006). Mitochondrial oxidative damage in aging and Alzheimer’s disease: Implications for mitochondrially targeted antioxidant therapeutics. J. Biomed. Biotechnol..

[94] Siddiqui A., Rivera-Sanchez S., Castro Mdel R., Acevedo-Torres K., Rane A., Torres-Ramos C.A., Nicholls D.G., Andersen J.K., Ayala-Torres S. (2012). Mitochondrial DNA damage is associated with reduced mitochondrial bioenergetics in Huntington’s disease. Free Radic. Biol. Med..

[95] Lin T.K., Cheng C.H., Chen S.D., Liou C.W., Huang C.R., Chuang Y.C. (2012). Mitochondrial dysfunction and oxidative stress promote apoptotic cell death in the striatum via cytochrome c/caspase-3 signaling cascade following chronic rotenone intoxication in rats. Int. J. Mol. Sci..

[96] Stutzbach L.D., Xie S.X., Naj A.C., Albin R., Gilman S., Lee V.M., Trojanowski J.Q., Devlin B., Schellenberg G.D., PSP Genetics Study Group (2013). The unfolded protein response is activated in disease-affected brain regions in progressive supranuclear palsy and Alzheimer’s disease. Acta Neuropathol. Commun..

[97] Leitman J., Barak B., Benyair R., Shenkman M., Ashery U., Hartl F.U., Lederkremer G.Z. (2014). ER stress-induced eIF2-alpha phosphorylation underlies sensitivity of striatal neurons to pathogenic huntingtin. PLoS ONE.

[98] Moreno J.A., Radford H., Peretti D., Steinert J.R., Verity N., Martin M.G., Halliday M., Morgan J., Dinsdale D., Ortori C.A. (2012). Sustained translational repression by eIF2alpha-P mediates prion neurodegeneration. Nature.

[99] Amodio G., Moltedo O., Fasano D., Zerillo L., Oliveti M., Di Pietro P., Faraonio R., Barone P., Pellecchia M.T., De Rosa A. (2019). PERK-Mediated Unfolded Protein Response Activation and Oxidative Stress in PARK20 Fibroblasts. Front. Neurosci..

[100] Kim J.K., Kang K.A., Ryu Y.S., Piao M.J., Han X., Oh M.C., Boo S.J., Jeong S.U., Jeong Y.J., Chae S. (2016). Induction of Endoplasmic Reticulum Stress via Reactive Oxygen Species Mediated by Luteolin in Melanoma Cells. Anticancer Res..

[101] Camargo L.L., Harvey A.P., Rios F.J., Tsiropoulou S., Da Silva R.N.O., Cao Z., Graham D., McMaster C., Burchmore R.J., Hartley R.C. (2018). Vascular Nox (NADPH Oxidase) Compartmentalization, Protein Hyperoxidation, and Endoplasmic Reticulum Stress Response in Hypertension. Hypertension.

[102] Xia Q., Feng X., Huang H., Du L., Yang X., Wang K. (2011). Gadolinium-induced oxidative stress triggers endoplasmic reticulum stress in rat cortical neurons. J. Neurochem..

[103] Kim M.J., Je A.R., Kim H.-J., Huh Y.H., Kweon H.-S. (2015). Coat protein I depletion-associated Golgi fragmentation in an Alzheimer’s disease model. Anim. Cells Syst..

[104] Fang C., Bourdette D., Banker G. (2012). Oxidative stress inhibits axonal transport: Implications for neurodegenerative diseases. Mol. Neurodegener..

[105] Ireland S.C., Huang H., Zhang J., Li J., Wang Y. (2020). Hydrogen peroxide induces Arl1 degradation and impairs Golgi-mediated trafficking. Mol. Biol. Cell.

[106] Nakagomi S., Barsoum M.J., Bossy-Wetzel E., Sutterlin C., Malhotra V., Lipton S.A. (2008). A Golgi fragmentation pathway in neurodegeneration. Neurobiol. Dis..

[107] Zhang J., Tan J., Hu Z., Chen C., Zeng L. (2019). HDAC6 Inhibition Protects against OGDR-Induced Golgi Fragmentation and Apoptosis. Oxid. Med. Cell. Longev..

[108] Zhong B., Hu Z., Tan J., Lu T., Lei Q., Chen C., Zeng L. (2015). Hsp20 Protects against Oxygen-Glucose Deprivation/Reperfusion-Induced Golgi Fragmentation and Apoptosis through Fas/FasL Pathway. Oxid. Med. Cell. Longev..

[109] Li T., You H., Mo X., He W., Tang X., Jiang Z., Chen S., Chen Y., Zhang J., Hu Z. (2016). GOLPH3 Mediated Golgi Stress Response in Modulating N2A Cell Death upon Oxygen-Glucose Deprivation and Reoxygenation Injury. Mol. Neurobiol..

[110] Miao F.F., Kong C.C., Wu Y., Fan L., Wang T.L. (2018). Golgi fragmentation induced by overactivated cyclin-dependent kinase 5 is associated with isoflurane-induced neurotoxicity. Neuroreport.

[111] Guo D., Xie W., Xiong P., Li H., Wang S., Chen G., Gao Y., Zhou J., Zhang Y., Bu G. (2018). Cyclin-dependent kinase 5-mediated phosphorylation of chloride intracellular channel 4 promotes oxidative stress-induced neuronal death. Cell Death Dis..

[112] Irato P., Santovito G. (2021). Enzymatic and Non-Enzymatic Molecules with Antioxidant Function. Antioxidants.

[113] Nordberg J., Arner E.S. (2001). Reactive oxygen species, antioxidants, and the mammalian thioredoxin system. Free Radic. Biol. Med..

[114] Scalbert A., Manach C., Morand C., Remesy C., Jimenez L. (2005). Dietary polyphenols and the prevention of diseases. Crit. Rev. Food Sci. Nutr..

[115] Spencer J.P., Abd El Mohsen M.M., Minihane A.M., Mathers J.C. (2008). Biomarkers of the intake of dietary polyphenols: Strengths, limitations and application in nutrition research. Br. J. Nutr..

[116] Pandey K.B., Rizvi S.I. (2009). Plant polyphenols as dietary antioxidants in human health and disease. Oxid. Med. Cell. Longev..

[117] Zhou Y., Zheng J., Li Y., Xu D.P., Li S., Chen Y.M., Li H.B. (2016). Natural Polyphenols for Prevention and Treatment of Cancer. Nutrients.

[118] Lotito S.B., Frei B. (2006). Consumption of flavonoid-rich foods and increased plasma antioxidant capacity in humans: Cause, consequence, or epiphenomenon?. Free Radic. Biol. Med..

[119] Manach C., Scalbert A., Morand C., Remesy C., Jimenez L. (2004). Polyphenols: Food sources and bioavailability. Am. J. Clin. Nutr..

[120] Gasic U., Ciric I., Pejcic T., Radenkovic D., Djordjevic V., Radulovic S., Tesic Z. (2020). Polyphenols as Possible Agents for Pancreatic Diseases. Antioxidants.

[121] Leri M., Scuto M., Ontario M.L., Calabrese V., Calabrese E.J., Bucciantini M., Stefani M. (2020). Healthy Effects of Plant Polyphenols: Molecular Mechanisms. Int. J. Mol. Sci..

[122] Lorenzo J.M., Mousavi Khaneghah A., Gavahian M., Marszalek K., Es I., Munekata P.E.S., Ferreira I., Barba F.J. (2019). Understanding the potential benefits of thyme and its derived products for food industry and consumer health: From extraction of value-added compounds to the evaluation of bioaccessibility, bioavailability, anti-inflammatory, and antimicrobial activities. Crit. Rev. Food Sci. Nutr..

[123] Ullah H., Khan H. (2018). Anti-Parkinson Potential of Silymarin: Mechanistic Insight and Therapeutic Standing. Front. Pharmacol..

[124] Palazzi L., Bruzzone E., Bisello G., Leri M., Stefani M., Bucciantini M., Polverino de Laureto P. (2018). Oleuropein aglycone stabilizes the monomeric alpha-synuclein and favours the growth of non-toxic aggregates. Sci. Rep..

[125] Kruk J., Aboul-Enein B.H., Duchnik E., Marchlewicz M. (2022). Antioxidative properties of phenolic compounds and their effect on oxidative stress induced by severe physical exercise. J. Physiol. Sci..

[126] Fiedor J., Burda K. (2014). Potential role of carotenoids as antioxidants in human health and disease. Nutrients.

[127] Maoka T. (2020). Carotenoids as natural functional pigments. J. Nat. Med..

[128] Jackson H., Braun C.L., Ernst H. (2008). The chemistry of novel xanthophyll carotenoids. Am. J. Cardiol..

[129] Kurashige M., Okimasu E., Inoue M., Utsumi K. (1990). Inhibition of oxidative injury of biological membranes by astaxanthin. Physiol. Chem. Phys. Med. NMR.

[130] Ohgami K., Shiratori K., Kotake S., Nishida T., Mizuki N., Yazawa K., Ohno S. (2003). Effects of astaxanthin on lipopolysaccharide-induced inflammation in vitro and in vivo. Investig. Ophthalmol. Vis. Sci..

[131] Ozawa Y., Sasaki M., Takahashi N., Kamoshita M., Miyake S., Tsubota K. (2012). Neuroprotective effects of lutein in the retina. Curr. Pharm. Des..

[132] Ciccone M.M., Cortese F., Gesualdo M., Carbonara S., Zito A., Ricci G., De Pascalis F., Scicchitano P., Riccioni G. (2013). Dietary intake of carotenoids and their antioxidant and anti-inflammatory effects in cardiovascular care. Mediat. Inflamm..

[133] Kabir M.T., Rahman M.H., Shah M., Jamiruddin M.R., Basak D., Al-Harrasi A., Bhatia S., Ashraf G.M., Najda A., El-Kott A.F. (2022). Therapeutic promise of carotenoids as antioxidants and anti-inflammatory agents in neurodegenerative disorders. Biomed. Pharmacother..

[134] Cho K.S., Shin M., Kim S., Lee S.B. (2018). Recent Advances in Studies on the Therapeutic Potential of Dietary Carotenoids in Neurodegenerative Diseases. Oxid. Med. Cell. Longev..

[135] Sanjeevi N., Lipsky L.M., Nansel T.R. (2019). Hyperglycemia and Carotenoid Intake Are Associated with Serum Carotenoids in Youth with Type 1 Diabetes. J. Acad. Nutr. Diet..

[136] Kowluru R.A., Menon B., Gierhart D.L. (2008). Beneficial effect of zeaxanthin on retinal metabolic abnormalities in diabetic rats. Investig. Ophthalmol. Vis. Sci..

[137] Althurwi H.N., Abdel-Rahman R.F., Soliman G.A., Ogaly H.A., Alkholifi F.K., Abd-Elsalam R.M., Alqasoumi S.I., Abdel-Kader M.S. (2022). Protective Effect of Beta-Carotene against Myeloperoxidase- Mediated Oxidative Stress and Inflammation in Rat Ischemic Brain Injury. Antioxidants.

[138] Ryan M.J., Dudash H.J., Docherty M., Geronilla K.B., Baker B.A., Haff G.G., Cutlip R.G., Alway S.E. (2010). Vitamin E and C supplementation reduces oxidative stress, improves antioxidant enzymes and positive muscle work in chronically loaded muscles of aged rats. Exp. Gerontol..

[139] Hosseinabadi M.B., Khanjani N., Norouzi P., Mirzaii M., Biganeh J., Nazarkhani F. (2020). Investigating the effects of vitamins E and C on oxidative stress and hematological parameters among power plant workers: A double-blind randomized controlled clinical trial. Toxicol. Ind. Health.

[140] Rizvi S., Raza S.T., Ahmed F., Ahmad A., Abbas S., Mahdi F. (2014). The role of vitamin e in human health and some diseases. Sultan Qaboos Univ. Med. J..

[141] Schirinzi T., Martella G., Imbriani P., Di Lazzaro G., Franco D., Colona V.L., Alwardat M., Sinibaldi Salimei P., Mercuri N.B., Pierantozzi M. (2019). Dietary Vitamin E as a Protective Factor for Parkinson’s Disease: Clinical and Experimental Evidence. Front. Neurol..

[142] Etminan M., Gill S.S., Samii A. (2005). Intake of vitamin E, vitamin C, and carotenoids and the risk of Parkinson’s disease: A meta-analysis. Lancet Neurol..

[143] Ricciarelli R., Argellati F., Pronzato M.A., Domenicotti C. (2007). Vitamin E and neurodegenerative diseases. Mol. Aspects Med..

[144] Lee K.H., Kim U.J., Cha M., Lee B.H. (2021). Chronic Treatment of Ascorbic Acid Leads to Age-Dependent Neuroprotection against Oxidative Injury in Hippocampal Slice Cultures. Int. J. Mol. Sci..

[145] Shen L., Ji H.F. (2012). Vitamin E: Supplement versus diet in neurodegenerative diseases. Trends Mol. Med..

[146] Taghizadeh M., Tamtaji O.R., Dadgostar E., Daneshvar Kakhaki R., Bahmani F., Abolhassani J., Aarabi M.H., Kouchaki E., Memarzadeh M.R., Asemi Z. (2017). The effects of omega-3 fatty acids and vitamin E co-supplementation on clinical and metabolic status in patients with Parkinson’s disease: A randomized, double-blind, placebo-controlled trial. Neurochem. Int..

[147] Marranzano M., Rosa R.L., Malaguarnera M., Palmeri R., Tessitori M., Barbera A.C. (2018). Polyphenols: Plant Sources and Food Industry Applications. Curr. Pharm. Des..

[148] Silva R.F.M., Pogacnik L. (2020). Polyphenols from Food and Natural Products: Neuroprotection and Safety. Antioxidants.

[149] Miller E.R., Pastor-Barriuso R., Dalal D., Riemersma R.A., Appel L.J., Guallar E. (2005). Meta-analysis: High-dosage vitamin E supplementation may increase all-cause mortality. Ann. Intern. Med..

[150] Bjelakovic G., Nikolova D., Gluud C. (2013). Meta-regression analyses, meta-analyses, and trial sequential analyses of the effects of supplementation with beta-carotene, vitamin A, and vitamin E singly or in different combinations on all-cause mortality: Do we have evidence for lack of harm?. PLoS ONE.

[151] Gong M., Bassi A. (2016). Carotenoids from microalgae: A review of recent developments. Biotechnol. Adv..

[152] Ryter S.W., Kim H.P., Hoetzel A., Park J.W., Nakahira K., Wang X., Choi A.M. (2007). Mechanisms of cell death in oxidative stress. Antioxid. Redox Signal..

[153] Xiang X., Kwame A.W., Qing Y., Li S., Wang M., Ren J. (2023). Natural antioxidants inhibit oxidative stress-induced changes in the morphology and motility of cells. Food Biosci..

[154] Martemucci G., Costagliola C., Mariano M., D’andrea L., Napolitano P., D’Alessandro A.G. (2022). Free Radical Properties, Source and Targets, Antioxidant Consumption and Health. Oxygen.

[155] Yahya R.A.M., Azab A.E., Shkal K.E.M. (2019). Effects of Copper Oxide and/or Zinc Oxide Nanoparticles on Oxidative Damage and Antioxidant Defense System in Male Albino Rats. EASJ Pharm. Pharmacol..

[156] Liu T., Xiao B., Xiang F., Tan J., Chen Z., Zhang X., Wu C., Mao Z., Luo G., Chen X. (2020). Ultrasmall copper-based nanoparticles for reactive oxygen species scavenging and alleviation of inflammation related diseases. Nat. Commun..

[157] Biswas S.K. (2016). Does the Interdependence between Oxidative Stress and Inflammation Explain the Antioxidant Paradox?. Oxid. Med. Cell. Longev..

[158] Gu Y., Dee C.M., Shen J. (2011). Interaction of free radicals, matrix metalloproteinases and caveolin-1 impacts blood-brain barrier permeability. Front. Biosci. (Schol. Ed.).

[159] Anderson G., Maes M. (2014). Oxidative/nitrosative stress and immuno-inflammatory pathways in depression: Treatment implications. Curr. Pharm. Des..

[160] Oliveira A.I., Pinho C., Sarmento B., Dias A.C.P. (2022). Quercetin-biapigenin nanoparticles are effective to penetrate the blood-brain barrier. Drug Deliv. Transl. Res..

[161] Li Y., Zhang X., Qi Z., Guo X., Liu X., Shi W., Liu Y., Du L. (2020). The enhanced protective effects of salvianic acid A: A functionalized nanoparticles against ischemic stroke through increasing the permeability of the blood-brain barrier. Nano Res..

[162] Adhikari A., Mondal S., Das M., Biswas P., Pal U., Darbar S., Bhattacharya S.S., Pal D., Saha-Dasgupta T., Das A.K. (2021). Incorporation of a Biocompatible Nanozyme in Cellular Antioxidant Enzyme Cascade Reverses Huntington’s Like Disorder in Preclinical Model. Adv. Healthc. Mater..

[163] Zhang B., Zhao Y., Guo K., Tian H., Wang C., Wang R., Chen Y., Chen X., Zheng H., Gao B. (2023). Macromolecular nanoparticles to attenuate both reactive oxygen species and inflammatory damage for treating Alzheimer’s disease. Bioeng. Transl. Med..

[164] Narendra Kumar S.K. (2016). Unique Properties (Essentials in Nanoscience and Nanotechnology).

[165] Rao C.N.R., Müller A.M., Cheetham A.K. (2004). The Chemistry of Nanomaterials: Synthesis, Properties and Applications.

[166] Patra M.K., Manzoor K., Vadera S.R., Kumar N. (2011). Functional Nanomaterials for Sensors and Display Applications, Encyclopedia of Nanoscience and Nanotechnology.

[167] Prabha S., Arya G., Chandra R., Ahmed B., Nimesh S. (2016). Effect of size on biological properties of nanoparticles employed in gene delivery. Artif. Cells Nanomed. Biotechnol..

[168] Song G., Petschauer J.S., Madden A.J., Zamboni W.C. (2014). Nanoparticles and the mononuclear phagocyte system: Pharmacokinetics and applications for inflammatory diseases. Curr. Rheumatol. Rev..

[169] Suk J.S., Xu Q., Kim N., Hanes J., Ensign L.M. (2016). PEGylation as a strategy for improving nanoparticle-based drug and gene delivery. Adv. Drug Deliv. Rev..

[170] Tenchov R., Bird R., Curtze A.E., Zhou Q. (2021). Lipid Nanoparticles horizontal line from Liposomes to mRNA Vaccine Delivery, a Landscape of Research Diversity and Advancement. ACS Nano.

[171] Sandhu K.K., McIntosh C.M., Simard J.M., Smith S.W., Rotello V.M. (2002). Gold nanoparticle-mediated transfection of mammalian cells. Bioconjug. Chem..

[172] Niidome T., Nakashima K., Takahashi H., Niidome Y. (2004). Preparation of primary amine-modified gold nanoparticles and their transfection ability into cultivated cells. Chem. Commun..

[173] De Jong W.H., Hagens W.I., Krystek P., Burger M.C., Sips A.J., Geertsma R.E. (2008). Particle size-dependent organ distribution of gold nanoparticles after intravenous administration. Biomaterials.

[174] Fang C., Shi B., Pei Y.Y., Hong M.H., Wu J., Chen H.Z. (2006). In vivo tumor targeting of tumor necrosis factor-alpha-loaded stealth nanoparticles: Effect of MePEG molecular weight and particle size. Eur. J. Pharm. Sci..

[175] Zhang G., Yang Z., Lu W., Zhang R., Huang Q., Tian M., Li L., Liang D., Li C. (2009). Influence of anchoring ligands and particle size on the colloidal stability and in vivo biodistribution of polyethylene glycol-coated gold nanoparticles in tumor-xenografted mice. Biomaterials.

[176] Stolnik S., Heald C.R., Neal J., Garnett M.C., Davis S.S., Illum L., Purkis S.C., Barlow R.J., Gellert P.R. (2001). Polylactide-poly(ethylene glycol) micellar-like particles as potential drug carriers: Production, colloidal properties and biological performance. J. Drug Target..

[177] Shenoy D.B., Amiji M.M. (2005). Poly(ethylene oxide)-modified poly(epsilon-caprolactone) nanoparticles for targeted delivery of tamoxifen in breast cancer. Int. J. Pharm..

[178] Lee H., Fonge H., Hoang B., Reilly R.M., Allen C. (2010). The effects of particle size and molecular targeting on the intratumoral and subcellular distribution of polymeric nanoparticles. Mol. Pharm..

[179] He C., Hu Y., Yin L., Tang C., Yin C. (2010). Effects of particle size and surface charge on cellular uptake and biodistribution of polymeric nanoparticles. Biomaterials.

[180] Yang N.J., Hinner M.J. (2015). Getting across the cell membrane: An overview for small molecules, peptides, and proteins. Methods Mol. Biol..

[181] Yanagishita M., Hascall V.C. (1992). Cell surface heparan sulfate proteoglycans. J. Biol. Chem..

[182] Mislick K.A., Baldeschwieler J.D. (1996). Evidence for the role of proteoglycans in cation-mediated gene transfer. Proc. Natl. Acad. Sci. USA.

[183] Kichler A., Mason A.J., Bechinger B. (2006). Cationic amphipathic histidine-rich peptides for gene delivery. Biochim. Biophys. Acta.

[184] Rejman J., Oberle V., Zuhorn I.S., Hoekstra D. (2004). Size-dependent internalization of particles via the pathways of clathrin- and caveolae-mediated endocytosis. Biochem. J..

[185] Gratton S.E., Ropp P.A., Pohlhaus P.D., Luft J.C., Madden V.J., Napier M.E., DeSimone J.M. (2008). The effect of particle design on cellular internalization pathways. Proc. Natl. Acad. Sci. USA.

[186] Panyam J., Labhasetwar V. (2003). Biodegradable nanoparticles for drug and gene delivery to cells and tissue. Adv. Drug Deliv. Rev..

[187] Shu X.Z., Zhu K.J. (2000). A novel approach to prepare tripolyphosphate/chitosan complex beads for controlled release drug delivery. Int. J. Pharm..

[188] Song C., Labhasetwar V., Cui X., Underwood T., Levy R.J. (1998). Arterial uptake of biodegradable nanoparticles for intravascular local drug delivery: Results with an acute dog model. J. Control. Release.

[189] Kus-Liskiewicz M., Fickers P., Ben Tahar I. (2021). Biocompatibility and Cytotoxicity of Gold Nanoparticles: Recent Advances in Methodologies and Regulations. Int. J. Mol. Sci..

[190] Ko W.C., Wang S.J., Hsiao C.Y., Hung C.T., Hsu Y.J., Chang D.C., Hung C.F. (2022). Pharmacological Role of Functionalized Gold Nanoparticles in Disease Applications. Molecules.

[191] Hajam Y.A., Rani R., Ganie S.Y., Sheikh T.A., Javaid D., Qadri S.S., Pramodh S., Alsulimani A., Alkhanani M.F., Harakeh S. (2022). Oxidative Stress in Human Pathology and Aging: Molecular Mechanisms and Perspectives. Cells.

[192] Kurutas E.B. (2016). The importance of antioxidants which play the role in cellular response against oxidative/nitrosative stress: Current state. Nutr. J..

[193] Zuhrotun A., Oktaviani D.J., Hasanah A.N. (2023). Biosynthesis of Gold and Silver Nanoparticles Using Phytochemical Compounds. Molecules.

[194] Xue J., Liu T., Liu Y., Jiang Y., Seshadri V.D.D., Mohan S.K., Ling L. (2019). Neuroprotective effect of biosynthesised gold nanoparticles synthesised from root extract of *Paeonia moutan* against Parkinson disease–In vitro & In vivo model. J. Photochem. Photobiol. B.

[195] Wang G., Dai J., Lu X. (2020). *Scutellaria barbata* Leaf Extract Mediated Gold Nanoparticles for Alzheimer’s Disease Treatment by Metal-Induced Amyloid β Aggregation Inhibition. J. Clust. Sci..

[196] Hardbower D.M., de Sablet T., Chaturvedi R., Wilson K.T. (2013). Chronic inflammation and oxidative stress: The smoking gun for Helicobacter pylori-induced gastric cancer?. Gut Microbes.

[197] Hazra S., Aziz A., Sharma S. (2022). Identification and screening of potential inhibitors obtained from *Plumeria rubra* L. compounds against type 2 diabetes mellitus. J. Biomol. Struct. Dyn..

[198] Hazra S., Singh R. (2021). Plants with Wound Healing Activity: A Review of Recent Clinical Trials. Int. J. Ayurvedic Med..

[199] Qiu X., Wu Y., Zhang D., Zhang H., Yu A., Li Z. (2021). Roles of Oxidative Stress and Raftlin in Wound Healing Under Negative-Pressure Wound Therapy. Clin. Cosmet. Investig. Dermatol..

[200] Liu Y., Kim S., Kim Y.J., Perumalsamy H., Lee S., Hwang E., Yi T.H. (2019). Green synthesis of gold nanoparticles using *Euphrasia officinalis* leaf extract to inhibit lipopolysaccharide-induced inflammation through NF-kappaB and JAK/STAT pathways in RAW 264.7 macrophages. Int. J. Nanomed..

[201] Leu J.G., Chen S.A., Chen H.M., Wu W.M., Hung C.F., Yao Y.D., Tu C.S., Liang Y.J. (2012). The effects of gold nanoparticles in wound healing with antioxidant epigallocatechin gallate and alpha-lipoic acid. Nanomedicine.

[202] Reuter S., Gupta S.C., Chaturvedi M.M., Aggarwal B.B. (2010). Oxidative stress, inflammation, and cancer: How are they linked?. Free Radic. Biol. Med..

[203] Singh R., Lillard J.W. (2009). Nanoparticle-based targeted drug delivery. Exp. Mol. Pathol..

[204] Ravi R., Zeyaullah M., Ghosh S., Khan Warsi M., Baweja R., AlShahrani A.M., Mishra A., Ahmad R. (2022). Use of gold nanoparticle-silibinin conjugates: A novel approach against lung cancer cells. Front. Chem..

[205] Yadav D., Nara S. (2023). Nanozymes for Neurodegenerative Diseases. Advances in Biological Sciences Research, Proceedings of the Conference BioSangam 2022: Emerging Trends in Biotechnology (BIOSANGAM 2022).

[206] Lou-Franco J., Das B., Elliott C., Cao C. (2020). Gold Nanozymes: From Concept to Biomedical Applications. Nanomicro. Lett..

[207] Zhang Y., Li S., Liu H., Long W., Zhang X.D. (2020). Enzyme-Like Properties of Gold Clusters for Biomedical Application. Front. Chem..

[208] Jiang Y., Kang Y., Liu J., Yin S., Huang Z., Shao L. (2022). Nanomaterials alleviating redox stress in neurological diseases: Mechanisms and applications. J. Nanobiotechnol..

[209] Dos Santos Tramontin N., da Silva S., Arruda R., Ugioni K.S., Canteiro P.B., de Bem Silveira G., Mendes C., Silveira P.C.L., Muller A.P. (2020). Gold Nanoparticles Treatment Reverses Brain Damage in Alzheimer’s Disease Model. Mol. Neurobiol..

[210] Mehanna E.T., Kamel B.S.A., Abo-Elmatty D.M., Elnabtity S.M., Mahmoud M.B., Abdelhafeez M.M., Sabry S.A.A. (2022). Effect of gold nanoparticles shape and dose on immunological, hematological, inflammatory, and antioxidants parameters in male rabbit. Vet. World.

[211] Wang P., Xie K., Wang C., Bi J. (2014). Oxidative stress induced by lipid peroxidation is related with inflammation of demyelination and neurodegeneration in multiple sclerosis. Eur. Neurol..

[212] Hou K., Zhao J., Wang H., Li B., Li K., Shi X., Wan K., Ai J., Lv J., Wang D. (2020). Chiral gold nanoparticles enantioselectively rescue memory deficits in a mouse model of Alzheimer’s disease. Nat. Commun..

[213] Dulamea A.O. (2017). The contribution of oligodendrocytes and oligodendrocyte progenitor cells to central nervous system repair in multiple sclerosis: Perspectives for remyelination therapeutic strategies. Neural Regen. Res..

[214] Spaas J., van Veggel L., Schepers M., Tiane A., van Horssen J., Wilson D.M., Moya P.R., Piccart E., Hellings N., Eijnde B.O. (2021). Oxidative stress and impaired oligodendrocyte precursor cell differentiation in neurological disorders. Cell. Mol. Life Sci..

[215] Robinson A.P., Zhang J.Z., Titus H.E., Karl M., Merzliakov M., Dorfman A.R., Karlik S., Stewart M.G., Watt R.K., Facer B.D. (2020). Nanocatalytic activity of clean-surfaced, faceted nanocrystalline gold enhances remyelination in animal models of multiple sclerosis. Sci. Rep..

[216] Johnson P., Krishnan V., Loganathan C., Govindhan K., Raji V., Sakayanathan P., Vijayan S., Sathishkumar P., Palvannan T. (2018). Rapid biosynthesis of *Bauhinia variegata* flower extract-mediated silver nanoparticles: An effective antioxidant scavenger and alpha-amylase inhibitor. Artif. Cells Nanomed. Biotechnol..

[217] Quinteros M.A., Cano Aristizabal V., Dalmasso P.R., Paraje M.G., Paez P.L. (2016). Oxidative stress generation of silver nanoparticles in three bacterial genera and its relationship with the antimicrobial activity. Toxicol. Vitr..

[218] Buttacavoli M., Albanese N.N., Di Cara G., Alduina R., Faleri C., Gallo M., Pizzolanti G., Gallo G., Feo S., Baldi F. (2018). Anticancer activity of biogenerated silver nanoparticles: An integrated proteomic investigation. Oncotarget.

[219] Sengani M., Bavithra V., Banerjee M., Choudhury A.A., Chakraborty S., Ramasubbu K., Rajeswari V.D., Al Obaid S., Alharbi S.A., Subramani B. (2022). Evaluation of the anti-diabetic effect of biogenic silver nanoparticles and intervention in PPARgamma gene regulation. Environ. Res..

[220] Anwar S., Almatroodi S.A., Almatroudi A., Allemailem K.S., Joseph R.J., Khan A.A., Alrumaihi F., Alsahli M.A., Husain Rahmani A. (2021). Biosynthesis of silver nanoparticles using *Tamarix articulata* leaf extract: An effective approach for attenuation of oxidative stress mediated diseases. Int. J. Food Prop..

[221] Kup F.O., Coskuncay S., Duman F. (2020). Biosynthesis of silver nanoparticles using leaf extract of *Aesculus hippocastanum* (horse chestnut): Evaluation of their antibacterial, antioxidant and drug release system activities. Mater. Sci. Eng. C Mater. Biol. Appl..

[222] Galdiero S., Falanga A., Vitiello M., Cantisani M., Marra V., Galdiero M. (2011). Silver nanoparticles as potential antiviral agents. Molecules.

[223] Jeyaraj M., Sathishkumar G., Sivanandhan G., MubarakAli D., Rajesh M., Arun R., Kapildev G., Manickavasagam M., Thajuddin N., Premkumar K. (2013). Biogenic silver nanoparticles for cancer treatment: An experimental report. Colloids Surf. B Biointerfaces.

[224] Bazaka K., Jacob M.V. (2017). Effects of Iodine Doping on Optoelectronic and Chemical Properties of Polyterpenol Thin Films. Nanomaterials.

[225] Siddiqi K.S., Husen A., Rao R.A.K. (2018). A review on biosynthesis of silver nanoparticles and their biocidal properties. J. Nanobiotechnol..

[226] Dilshad E., Bibi M., Sheikh N.A., Tamrin K.F., Mansoor Q., Maqbool Q., Nawaz M. (2020). Synthesis of Functional Silver Nanoparticles and Microparticles with Modifiers and Evaluation of Their Antimicrobial, Anticancer, and Antioxidant Activity. J. Funct. Biomater..

[227] Gonzalez-Carter D.A., Leo B.F., Ruenraroengsak P., Chen S., Goode A.E., Theodorou I.G., Chung K.F., Carzaniga R., Shaffer M.S., Dexter D.T. (2017). Silver nanoparticles reduce brain inflammation and related neurotoxicity through induction of H(2)S-synthesizing enzymes. Sci. Rep..

[228] Kajita M., Hikosaka K., Iitsuka M., Kanayama A., Toshima N., Miyamoto Y. (2007). Platinum nanoparticle is a useful scavenger of superoxide anion and hydrogen peroxide. Free Radic. Res..

[229] Martin R., Menchon C., Apostolova N., Victor V.M., Alvaro M., Herance J.R., Garcia H. (2010). Nano-jewels in biology. Gold and platinum on diamond nanoparticles as antioxidant systems against cellular oxidative stress. ACS Nano.

[230] Gatto F., Moglianetti M., Pompa P.P., Bardi G. (2018). Platinum Nanoparticles Decrease Reactive Oxygen Species and Modulate Gene Expression without Alteration of Immune Responses in THP-1 Monocytes. Nanomaterials.

[231] Huang X., He D., Pan Z., Luo G., Deng J. (2021). Reactive-oxygen-species-scavenging nanomaterials for resolving inflammation. Mater. Today Bio.

[232] Wu Y., Song Z., Deng G., Jiang K., Wang H., Zhang X., Han H. (2021). Gastric Acid Powered Nanomotors Release Antibiotics for In Vivo Treatment of Helicobacter pylori Infection. Small.

[233] Ismail N.A.S., Lee J.X., Yusof F. (2022). Platinum Nanoparticles: The Potential Antioxidant in the Human Lung Cancer Cells. Antioxidants.

[234] Takamiya M., Miyamoto Y., Yamashita T., Deguchi K., Ohta Y., Abe K. (2012). Strong neuroprotection with a novel platinum nanoparticle against ischemic stroke- and tissue plasminogen activator-related brain damages in mice. Neuroscience.

[235] Nellore J., Pauline C., Amarnath K. (2013). Bacopa monnieri Phytochemicals Mediated Synthesis of Platinum Nanoparticles and Its Neurorescue Effect on 1-Methyl 4-Phenyl 1,2,3,6 Tetrahydropyridine-Induced Experimental Parkinsonism in Zebrafish. J. Neurodegener. Dis..

[236] Lamb D.J., Tickner M.L., Hourani S.M., Ferns G.A. (2005). Dietary copper supplements modulate aortic superoxide dismutase, nitric oxide and atherosclerosis. Int. J. Exp. Pathol..

[237] Lin C., Guo X., Mo F., Sun D. (2023). Different Dimensional Copper-Based Metal-Organic Frameworks with Enzyme-Mimetic Activity for Antibacterial Therapy. Int. J. Mol. Sci..

[238] Gottfredsen R.H., Larsen U.G., Enghild J.J., Petersen S.V. (2013). Hydrogen peroxide induce modifications of human extracellular superoxide dismutase that results in enzyme inhibition. Redox Biol..

[239] Schacter L.P., DelVillano B.C., Gordon E.M., Klein B.L. (1985). Red cell superoxide dismutase and sickle cell anemia symptom severity. Am. J. Hematol..

[240] Scott M.D., Eaton J.W., Kuypers F.A., Chiu D.T., Lubin B.H. (1989). Enhancement of erythrocyte superoxide dismutase activity: Effects on cellular oxidant defense. Blood.

[241] Shkal K.E.M., Azab A.E., Attia A.M., El-Banna S.G., Yahya R.A.M. (2020). Zinc oxide nanoparticles attenuate the oxidative damage and disturbance in antioxidant defense system induced by cyclophosphamide in male albino rats. Insights Biol. Med..

[242] Soren S., Kumar S., Mishra S., Jena P.K., Verma S.K., Parhi P. (2018). Evaluation of antibacterial and antioxidant potential of the zinc oxide nanoparticles synthesized by aqueous and polyol method. Microb. Pathog..

[243] Nagajyothi P.C., Cha S.J., Yang I.J., Sreekanth T.V.M., Kim K.J., Shin H.M. (2015). Antioxidant and anti-inflammatory activities of zinc oxide nanoparticles synthesized using *Polygala tenuifolia* root extract. J. Photochem. Photobiol. B Biol..

[244] Saleemi M.A., Alallam B., Yong Y.K., Lim V. (2022). Synthesis of Zinc Oxide Nanoparticles with Bioflavonoid Rutin: Characterisation, Antioxidant and Antimicrobial Activities and In Vivo Cytotoxic Effects on *Artemia* Nauplii. Antioxidants.

[245] Vera J., Herrera W., Hermosilla E., Díaz M., Parada J., Seabra A.B., Tortella G., Pesenti H., Ciudad G., Rubilar O. (2023). Antioxidant Activity as an Indicator of the Efficiency of Plant Extract-Mediated Synthesis of Zinc Oxide Nanoparticles. Antioxidants.

[246] Abdulmalek S., Nasef M., Awad D., Balbaa M. (2021). Protective Effect of Natural Antioxidant, Curcumin Nanoparticles, and Zinc Oxide Nanoparticles against Type 2 Diabetes-Promoted Hippocampal Neurotoxicity in Rats. Pharmaceutics.

[247] Bottino C.M.C., Castro C.C., Gomes R.L.E., Buchpiguel C.A., Marchetti R.L., Neto M.R.L. (2002). Volumetric MRI Measurements Can Differentiate Alzheimer’s Disease, Mild Cognitive Impairment, and Normal Aging. Int. Psychogeriatr..

[248] Al-Radadi N.S., Abdullah, Faisal S., Alotaibi A., Ullah R., Hussain T., Rizwan M., Saira, Zaman N., Iqbal M. (2022). Zingiber officinale driven bioproduction of ZnO nanoparticles and their anti-inflammatory, anti-diabetic, anti-Alzheimer, anti-oxidant, and anti-microbial applications. Inorg. Chem. Commun..

[249] Dappula S.S., Kandrakonda Y.R., Shaik J.B., Mothukuru S.L., Lebaka V.R., Mannarapu M., Amooru G.D. (2023). Biosynthesis of zinc oxide nanoparticles using aqueous extract of *Andrographis alata*: Characterization, optimization and assessment of their antibacterial, antioxidant, antidiabetic and anti-Alzheimer’s properties. J. Mol. Struct..

[250] Hussein J., El-Naggar M.E., Anwar M., Latif Y.A., Khateeb S. (2019). Synthesis of docosahexaenoic acid–loaded zinc oxide nanoparticles as a promising treatment in neurotoxicity. Comp. Clin. Pathol..

[251] Alharthi N.S., Aldakheel F.M., Binshaya A.S. (2022). Inhibition of Glycogen Synthase Kinase and the Neuroprotective Function of Conjugated ZnO-Osthol Nanoparticles in Alzheimer’s Disease. Bioinorg. Chem. Appl..

[252] Mahendiran C., Ganesan R., Gedanken A. (2009). Sonoelectrochemical Synthesis of Metallic Aluminum Nanoparticles. Eur. J. Inorg. Chem..

[253] Bokhary K.A., Maqsood F., Amina M., Aldarwesh A., Mofty H.K., Al-Yousef H.M. (2022). Grapefruit Extract-Mediated Fabrication of Photosensitive Aluminum Oxide Nanoparticle and Their Antioxidant and Anti-Inflammatory Potential. Nanomaterials.

[254] Nawaz S., Nordell P., Hillborg H., Gedde U.W. (2012). Antioxidant activity in aluminium oxide—Poly(ethylene-co-butyl acrylate) nanocomposites. Polym. Degrad. Stab..

[255] Tuqa S., Kareem H.J., Nebras A. (2023). Synthesis and Biomedical Activity of Aluminium Oxide Nanoparticles by Laser Ablation Technique. Res. J. Pharm. Technol..

[256] Khalil I., Yehye W.A., Etxeberria A.E., Alhadi A.A., Dezfooli S.M., Julkapli N.B.M., Basirun W.J., Seyfoddin A. (2019). Nanoantioxidants: Recent Trends in Antioxidant Delivery Applications. Antioxidants.

[257] Bai S., Jiang H., Song Y., Zhu Y., Qin M., He C., Du G., Sun X. (2022). Aluminum nanoparticles deliver a dual-epitope peptide for enhanced anti-tumor immunotherapy. J. Control. Release.

[258] Guan B., Yan R., Li R., Zhang X. (2018). Selenium as a pleiotropic agent for medical discovery and drug delivery. Int. J. Nanomed..

[259] Monsen E.R. (2000). Dietary reference intakes for the antioxidant nutrients: Vitamin C, vitamin E, selenium, and carotenoids. J. Am. Diet. Assoc..

[260] Koller L.D., Exon J.H. (1986). The two faces of selenium-deficiency and toxicity—Are similar in animals and man. Can. J. Vet. Res..

[261] Muhammad I., Zhikun C., Ayaz M., Shah R., Wang W., Waleed A., Farhan I., Volkan G., Adem K., Abdulsamed K. (2023). Distribution of Selenium in Soils and Human Health. Selenium and Human Health.

[262] Rehman A., John P., Bhatti A. (2021). Biogenic Selenium Nanoparticles: Potential Solution to Oxidative Stress Mediated Inflammation in Rheumatoid Arthritis and Associated Complications. Nanomaterials.

[263] Turovsky E.A., Mal’tseva V.N., Sarimov R.M., Simakin A.V., Gudkov S.V., Plotnikov E.Y. (2022). Features of the cytoprotective effect of selenium nanoparticles on primary cortical neurons and astrocytes during oxygen-glucose deprivation and reoxygenation. Sci. Rep..

[264] Hadrup N., Loeschner K., Mandrup K., Ravn-Haren G., Frandsen H.L., Larsen E.H., Lam H.R., Mortensen A. (2019). Subacute oral toxicity investigation of selenium nanoparticles and selenite in rats. Drug Chem. Toxicol..

[265] Khalil H.M.A., Azouz R.A., Hozyen H.F., Aljuaydi S.H., AbuBakr H.O., Emam S.R., Al-Mokaddem A.K. (2022). Selenium nanoparticles impart robust neuroprotection against deltamethrin-induced neurotoxicity in male rats by reversing behavioral alterations, oxidative damage, apoptosis, and neuronal loss. Neurotoxicology.

[266] Ferro C., Florindo H.F., Santos H.A. (2021). Selenium Nanoparticles for Biomedical Applications: From Development and Characterization to Therapeutics. Adv. Healthc. Mater..

[267] Dominiak A., Wilkaniec A., Wroczynski P., Adamczyk A. (2016). Selenium in the Therapy of Neurological Diseases. Where is it Going?. Curr. Neuropharmacol..

[268] Pillai R., Uyehara-Lock J.H., Bellinger F.P. (2014). Selenium and selenoprotein function in brain disorders. IUBMB Life.

[269] Ashraf H., Cossu D., Ruberto S., Noli M., Jasemi S., Simula E.R., Sechi L.A. (2023). Latent Potential of Multifunctional Selenium Nanoparticles in Neurological Diseases and Altered Gut Microbiota. Materials.

[270] Yang L., Sun J., Xie W., Liu Y., Liu J. (2017). Dual-functional selenium nanoparticles bind to and inhibit amyloid beta fiber formation in Alzheimer’s disease. J. Mater. Chem. B.

[271] Yang L., Wang N., Zheng G. (2018). Enhanced Effect of Combining Chlorogenic Acid on Selenium Nanoparticles in Inhibiting Amyloid beta Aggregation and Reactive Oxygen Species Formation In Vitro. Nanoscale Res. Lett..

[272] Chen G., Yang F., Fan S., Jin H., Liao K., Li X., Liu G.B., Liang J., Zhang J., Xu J.F. (2022). Immunomodulatory roles of selenium nanoparticles: Novel arts for potential immunotherapy strategy development. Front. Immunol..

[273] Garza-Garcia J.J.O., Hernandez-Diaz J.A., Zamudio-Ojeda A., Leon-Morales J.M., Guerrero-Guzman A., Sanchez-Chipres D.R., Lopez-Velazquez J.C., Garcia-Morales S. (2022). The Role of Selenium Nanoparticles in Agriculture and Food Technology. Biol. Trace Elem. Res..

[274] Sharda S.S., Shukla A.K. (2021). Potential Therapeutic Applications of Nano-antioxidants.

[275] Yang L., Wang W., Chen J., Wang N., Zheng G. (2018). A comparative study of resveratrol and resveratrol-functional selenium nanoparticles: Inhibiting amyloid beta aggregation and reactive oxygen species formation properties. J. Biomed. Mater. Res. A.

[276] Wang Y., Luo W., Lin F., Liu W., Gu R. (2022). Epigallocatechin-3-gallate selenium nanoparticles for neuroprotection by scavenging reactive oxygen species and reducing inflammation. Front. Bioeng. Biotechnol..

[277] Huo X., Zhang Y., Jin X., Li Y., Zhang L. (2019). A novel synthesis of selenium nanoparticles encapsulated PLGA nanospheres with curcumin molecules for the inhibition of amyloid beta aggregation in Alzheimer’s disease. J. Photochem. Photobiol. B.

[278] Sun D., Zhang W., Yu Q., Chen X., Xu M., Zhou Y., Liu J. (2017). Chiral penicillamine-modified selenium nanoparticles enantioselectively inhibit metal-induced amyloid beta aggregation for treating Alzheimer’s disease. J. Colloid Interface Sci..

[279] Nirmala C., Sridevi M., Aishwarya A., Perara R., Sathiyanarayanan Y. (2023). Pharmacological Prospects of Morin Conjugated Selenium Nanoparticles—Evaluation of Antimicrobial, Antioxidant, Thrombolytic, and Anticancer Activities. BioNanoScience.

[280] Yin T., Yang L., Liu Y., Zhou X., Sun J., Liu J. (2015). Sialic acid (SA)-modified selenium nanoparticles coated with a high blood-brain barrier permeability peptide-B6 peptide for potential use in Alzheimer’s disease. Acta Biomater..

[281] Li W., Liu Z., Liu C., Guan Y., Ren J., Qu X. (2017). Manganese Dioxide Nanozymes as Responsive Cytoprotective Shells for Individual Living Cell Encapsulation. Angew. Chem. Int. Ed. Engl..

[282] Liu X., Wang Q., Zhao H., Zhang L., Su Y., Lv Y. (2012). BSA-templated MnO_2_ nanoparticles as both peroxidase and oxidase mimics. Analyst.

[283] Yao J., Cheng Y., Zhou M., Zhao S., Lin S., Wang X., Wu J., Li S., Wei H. (2018). ROS scavenging Mn_3_O_4_ nanozymes for in vivo anti-inflammation. Chem. Sci..

[284] Singh N., Savanur M.A., Srivastava S., D’Silva P., Mugesh G. (2019). A manganese oxide nanozyme prevents the oxidative damage of biomolecules without affecting the endogenous antioxidant system. Nanoscale.

[285] Liang S., Tian X., Wang C. (2022). Nanozymes in the Treatment of Diseases Caused by Excessive Reactive Oxygen Specie. J. Inflamm. Res..

[286] Park E., Li L.Y., He C., Abbasi A.Z., Ahmed T., Foltz W.D., O’Flaherty R., Zain M., Bonin R.P., Rauth A.M. (2023). Brain-Penetrating and Disease Site-Targeting Manganese Dioxide-Polymer-Lipid Hybrid Nanoparticles Remodel Microenvironment of Alzheimer’s Disease by Regulating Multiple Pathological Pathways. Adv. Sci..

[287] Kuthati Y., Busa P., Goutham Davuluri V.N., Wong C.S. (2019). Manganese Oxide Nanozymes Ameliorate Mechanical Allodynia in a Rat Model of Partial Sciatic Nerve-Transection Induced Neuropathic Pain. Int. J. Nanomed..

[288] Jafari S., Mahyad B., Hashemzadeh H., Janfaza S., Gholikhani T., Tayebi L. (2020). Biomedical Applications of TiO_2_ Nanostructures: Recent Advances. Int. J. Nanomed..

[289] Alijagic A., Gaglio D., Napodano E., Russo R., Costa C., Benada O., Kofroňová O., Pinsino A. (2020). Titanium dioxide nanoparticles temporarily influence the sea urchin immunological state suppressing inflammatory-relate gene transcription and boosting antioxidant metabolic activity. J. Hazard. Mater..

[290] Ajmal N., Saraswat K., Bakht M.A., Riadi Y., Ahsan M.J., Noushad M. (2019). Cost-effective and eco-friendly synthesis of titanium dioxide (TiO_2_) nanoparticles using fruit’s peel agro-waste extracts: Characterization, in vitro antibacterial, antioxidant activities. Green Chem. Lett. Rev..

[291] Gul H., Javed H.M.A., Awais M., Javaid M.Y., Khan M.I., Arif M., Alshahrani M.Y., Khalil R.M.A., Khan F.S., Galal A.M. (2022). TiO_2_ nanoparticles functionalized with marigold for antioxidant role to enhance the skin protection. Biomass Convers. Biorefinery.

[292] Pechanova O., Dayar E., Cebova M. (2020). Therapeutic Potential of Polyphenols-Loaded Polymeric Nanoparticles in Cardiovascular System. Molecules.

[293] Mbenga Y., Adeyemi J.O., Mthiyane D.M.N., Singh M., Onwudiwe D.C. (2023). Green synthesis, antioxidant and anticancer activities of TiO_2_ nanoparticles using aqueous extract of *Tulbhagia violacea*. Results Chem..

[294] Punitha V.N., Vijayakumar S., Sakthivel B., Praseetha P.K. (2020). Protection of neuronal cell lines, antimicrobial and photocatalytic behaviours of eco-friendly TiO_2_ nanoparticles. J. Environ. Chem. Eng..

[295] Cheng K., Peng S., Xu C., Sun S. (2009). Porous hollow Fe_3_O_4_ nanoparticles for targeted delivery and controlled release of cisplatin. J. Am. Chem. Soc..

[296] Chertok B., Moffat B.A., David A.E., Yu F., Bergemann C., Ross B.D., Yang V.C. (2008). Iron oxide nanoparticles as a drug delivery vehicle for MRI monitored magnetic targeting of brain tumors. Biomaterials.

[297] Maeng J.H., Lee D.H., Jung K.H., Bae Y.H., Park I.S., Jeong S., Jeon Y.S., Shim C.K., Kim W., Kim J. (2010). Multifunctional doxorubicin loaded superparamagnetic iron oxide nanoparticles for chemotherapy and magnetic resonance imaging in liver cancer. Biomaterials.

[298] Lu C.-H., Hsiao J.-K. (2023). Diagnostic and therapeutic roles of iron oxide nanoparticles in biomedicine. Tzu Chi Med. J..

[299] Lee H., Yu M.K., Park S., Moon S., Min J.J., Jeong Y.Y., Kang H.W., Jon S. (2007). Thermally cross-linked superparamagnetic iron oxide nanoparticles: Synthesis and application as a dual imaging probe for cancer in vivo. J. Am. Chem. Soc..

[300] Dadfar S.M., Roemhild K., Drude N.I., von Stillfried S., Knüchel R., Kiessling F., Lammers T. (2019). Iron oxide nanoparticles: Diagnostic, therapeutic and theranostic applications. Adv. Drug Deliv. Rev..

[301] Zhang Y., Wang Z., Li X., Wang L., Yin M., Wang L., Chen N., Fan C., Song H. (2016). Dietary Iron Oxide Nanoparticles Delay Aging and Ameliorate Neurodegeneration in *Drosophila*. Adv. Mater..

[302] Grau C.M., Greene L.A. (2012). Use of PC12 cells and rat superior cervical ganglion sympathetic neurons as models for neuroprotective assays relevant to Parkinson’s disease. Methods Mol. Biol..

[303] Xia Z., Gao M., Sheng P., Shen M., Zhao L., Gao L., Yan B. (2022). Fe_3_O_4_ Nanozymes Improve Neuroblast Differentiation and Blood-Brain Barrier Integrity of the Hippocampal Dentate Gyrus in D-Galactose-Induced Aged Mice. Int. J. Mol. Sci..

[304] Luo S., Ma C., Zhu M.Q., Ju W.N., Yang Y., Wang X. (2020). Application of Iron Oxide Nanoparticles in the Diagnosis and Treatment of Neurodegenerative Diseases with Emphasis on Alzheimer’s Disease. Front. Cell. Neurosci..

[305] Hashemian M., Ghasemi-Kasman M., Ghasemi S., Akbari A., Moalem-Banhangi M., Zare L., Ahmadian S.R. (2019). Fabrication and evaluation of novel quercetin-conjugated Fe_3_O_4_-beta-cyclodextrin nanoparticles for potential use in epilepsy disorder. Int. J. Nanomed..

[306] Kim J., Hong G., Mazaleuskaya L., Hsu J.C., Rosario-Berrios D.N., Grosser T., Cho-Park P.F., Cormode D.P. (2021). Ultrasmall Antioxidant Cerium Oxide Nanoparticles for Regulation of Acute Inflammation. ACS Appl. Mater. Interfaces.

[307] Rzigalinski B.A., Meehan K., Davis R.M., Xu Y., Miles W.C., Cohen C.A. (2006). Radical nanomedicine. Nanomedicine.

[308] Nelson B.C., Johnson M.E., Walker M.L., Riley K.R., Sims C.M. (2016). Antioxidant Cerium Oxide Nanoparticles in Biology and Medicine. Antioxidants.

[309] Korsvik C., Patil S., Seal S., Self W.T. (2007). Superoxide dismutase mimetic properties exhibited by vacancy engineered ceria nanoparticles. Chem. Commun..

[310] Patel P., Kansara K., Singh R., Shukla R.K., Singh S., Dhawan A., Kumar A. (2018). Cellular internalization and antioxidant activity of cerium oxide nanoparticles in human monocytic leukemia cells. Int. J. Nanomed..

[311] Gravina N., Ruso J.M., Mbeh D.A., Yahia L.H., Merhi Y., Sartuqui J., Messina P.V. (2015). Effect of ceria on the organization and bio-ability of anatase fullerene-like crystals. RSC Adv..

[312] Chigurupati S., Mughal M.R., Okun E., Das S., Kumar A., McCaffery M., Seal S., Mattson M.P. (2013). Effects of cerium oxide nanoparticles on the growth of keratinocytes, fibroblasts and vascular endothelial cells in cutaneous wound healing. Biomaterials.

[313] Dal Magro R., Vitali A., Fagioli S., Casu A., Falqui A., Formicola B., Taiarol L., Cassina V., Marrano C.A., Mantegazza F. (2021). Oxidative Stress Boosts the Uptake of Cerium Oxide Nanoparticles by Changing Brain Endothelium Microvilli Pattern. Antioxidants.

[314] Nele V., Tedeschi V., Campani V., Ciancio R., Angelillo A., Graziano S.F., De Rosa G., Secondo A. (2023). Cerium-Doped Self-Assembling Nanoparticles as a Novel Anti-Oxidant Delivery System Preserving Mitochondrial Function in Cortical Neurons Exposed to Ischemia-like Conditions. Antioxidants.

[315] Kim M.J., Rehman S.U., Amin F.U., Kim M.O. (2017). Enhanced neuroprotection of anthocyanin-loaded PEG-gold nanoparticles against Abeta(1-42)-induced neuroinflammation and neurodegeneration via the NF-(K)B /JNK/GSK3beta signaling pathway. Nanomedicine.

[316] Marcuzzo S., Isaia D., Bonanno S., Malacarne C., Cavalcante P., Zacheo A., Laquintana V., Denora N., Sanavio B., Salvati E. (2019). FM19G11-Loaded Gold Nanoparticles Enhance the Proliferation and Self-Renewal of Ependymal Stem Progenitor Cells Derived from ALS Mice. Cells.

[317] Zhang X., Li Y., Hu Y. (2020). Green synthesis of silver nanoparticles and their preventive effect in deficits in recognition and spatial memory in sporadic Alzheimer’s rat model. Colloids Surf. A Physicochem. Eng. Asp..

[318] Hao C., Qu A., Xu L., Sun M., Zhang H., Xu C., Kuang H. (2019). Chiral Molecule-mediated Porous Cu *_x_*O Nanoparticle Clusters with Antioxidation Activity for Ameliorating Parkinson’s Disease. J. Am. Chem. Soc..

[319] Ma M., Liu Z., Gao N., Pi Z., Du X., Ren J., Qu X. (2020). Self-Protecting Biomimetic Nanozyme for Selective and Synergistic Clearance of Peripheral Amyloid-beta in an Alzheimer’s Disease Model. J. Am. Chem. Soc..

[320] Vilella A., Belletti D., Sauer A.K., Hagmeyer S., Sarowar T., Masoni M., Stasiak N., Mulvihill J.J.E., Ruozi B., Forni F. (2018). Reduced plaque size and inflammation in the APP23 mouse model for Alzheimer’s disease after chronic application of polymeric nanoparticles for CNS targeted zinc delivery. J. Trace Elem. Med. Biol..

[321] Abd Elmonem H.A., Morsi R.M., Mansour D.S., El-Sayed E.-S.R. (2023). Myco-fabricated ZnO nanoparticles ameliorate neurotoxicity in mice model of Alzheimer’s disease via acetylcholinesterase inhibition and oxidative stress reduction. BioMetals.

[322] Abozaid O.A.R., Sallam M.W., El-Sonbaty S., Aziza S., Emad B., Ahmed E.S.A. (2022). Resveratrol-Selenium Nanoparticles Alleviate Neuroinflammation and Neurotoxicity in a Rat Model of Alzheimer’s Disease by Regulating Sirt1/miRNA-134/GSK3beta Expression. Biol. Trace Elem. Res..

[323] Li C., Wang N., Zheng G., Yang L. (2021). Oral Administration of Resveratrol-Selenium-Peptide Nanocomposites Alleviates Alzheimer’s Disease-like Pathogenesis by Inhibiting Aβ Aggregation and Regulating Gut Microbiota. ACS Appl. Mater. Interfaces.

[324] Salaramoli S., Amiri H., Joshaghani H.R., Hosseini M., Hashemy S.I. (2023). Bio-synthesized selenium nanoparticles ameliorate Brain oxidative stress in Parkinson disease rat models. Metab. Brain Dis..

[325] Mohamed K.M., Abdelfattah M.S., El-khadragy M., Al-Megrin W.A., Fehaid A., Kassab R.B., Moneim A.E.A. (2023). Rutin-loaded selenium nanoparticles modulated the redox status, inflammatory, and apoptotic pathways associated with pentylenetetrazole-induced epilepsy in mice. Green Process. Synth..

[326] Amanzadeh Jajin E., Esmaeili A., Rahgozar S., Noorbakhshnia M. (2020). Quercetin-Conjugated Superparamagnetic Iron Oxide Nanoparticles Protect AlCl(3)-Induced Neurotoxicity in a Rat Model of Alzheimer’s Disease via Antioxidant Genes, APP Gene, and miRNA-101. Front. Neurosci..

[327] Chen X., Guo X., Hao S., Yang T., Wang J. (2022). Iron oxide nanoparticles-loaded hyaluronic acid nanogels for MRI-aided Alzheimer’s disease theranostics. Arab. J. Chem..

[328] Umarao P., Bose S., Bhattacharyya S., Kumar A., Jain S. (2016). Neuroprotective Potential of Superparamagnetic Iron Oxide Nanoparticles along with Exposure to Electromagnetic Field in 6-OHDA Rat Model of Parkinson’s Disease. J. Nanosci. Nanotechnol..

[329] Kwon H.J., Cha M.Y., Kim D., Kim D.K., Soh M., Shin K., Hyeon T., Mook-Jung I. (2016). Mitochondria-Targeting Ceria Nanoparticles as Antioxidants for Alzheimer’s Disease. ACS Nano.

[330] Sundararajan V., Venkatasubbu G.D., Sheik Mohideen S. (2021). Investigation of therapeutic potential of cerium oxide nanoparticles in Alzheimer’s disease using transgenic *Drosophila*. 3 Biotech.

[331] Bailey Z.S., Nilson E., Bates J.A., Oyalowo A., Hockey K.S., Sajja V.S.S.S., Thorpe C., Rogers H., Dunn B., Frey A.S. (2020). Cerium oxide nanoparticles improve outcome after in vitro and in vivo mild traumatic brain injury. J. Neurotrauma.

[332] Padmanabhan P., Palanivel M., Kumar A., Mathe D., Radda G.K., Lim K.L., Gulyas B. (2020). Nanotheranostic agents for neurodegenerative diseases. Emerg. Top. Life Sci..

[333] Ladju R.B., Ulhaq Z.S., Soraya G.V. (2022). Nanotheranostics: A powerful next-generation solution to tackle hepatocellular carcinoma. World J. Gastroenterol..

[334] Zhang P., Zhang L., Qin Z., Hua S., Guo Z., Chu C., Lin H., Zhang Y., Li W., Zhang X. (2018). Genetically Engineered Liposome-like Nanovesicles as Active Targeted Transport Platform. Adv. Mater..

[335] Kumar A., Chaudhary R.K., Singh R., Singh S.P., Wang S.Y., Hoe Z.Y., Pan C.T., Shiue Y.L., Wei D.Q., Kaushik A.C. (2020). Nanotheranostic Applications for Detection and Targeting Neurodegenerative Diseases. Front. Neurosci..

[336] Jeelani S., Reddy R.C., Maheswaran T., Asokan G.S., Dany A., Anand B. (2014). Theranostics: A treasured tailor for tomorrow. J. Pharm. Bioallied Sci..

[337] Muthu M.S., Leong D.T., Mei L., Feng S.S. (2014). Nanotheranostics–application and further development of nanomedicine strategies for advanced theranostics. Theranostics.

[338] Mulkey D.K., Henderson R.A., Ritucci N.A., Putnam R.W., Dean J.B. (2004). Oxidative stress decreases pH_i_ and Na^+^/H^+^ exchange and increases excitability of solitary complex neurons from rat brain slices. Am. J. Physiol. Cell Physiol..

[339] Pu H.L., Chiang W.L., Maiti B., Liao Z.X., Ho Y.C., Shim M.S., Chuang E.Y., Xia Y., Sung H.W. (2014). Nanoparticles with dual responses to oxidative stress and reduced ph for drug release and anti-inflammatory applications. ACS Nano.

[340] Yu P., Yu H., Guo C., Cui Z., Chen X., Yin Q., Zhang P., Yang X., Cui H., Li Y. (2015). Reversal of doxorubicin resistance in breast cancer by mitochondria-targeted pH-responsive micelles. Acta Biomater..

[341] Lin H., Chen Y., Shi J. (2018). Nanoparticle-triggered in situ catalytic chemical reactions for tumour-specific therapy. Chem. Soc. Rev..

[342] Zhu J., Wang G., Alves C.S., Tomas H., Xiong Z., Shen M., Rodrigues J., Shi X. (2018). Multifunctional Dendrimer-Entrapped Gold Nanoparticles Conjugated with Doxorubicin for pH-Responsive Drug Delivery and Targeted Computed Tomography Imaging. Langmuir.

[343] Xiao J., Zhang G., Xu R., Chen H., Wang H., Tian G., Wang B., Yang C., Bai G., Zhang Z. (2019). A pH-responsive platform combining chemodynamic therapy with limotherapy for simultaneous bioimaging and synergistic cancer therapy. Biomaterials.

[344] Song X.R., Li S.H., Dai J., Song L., Huang G., Lin R., Li J., Liu G., Yang H.H. (2017). Polyphenol-Inspired Facile Construction of Smart Assemblies for ATP- and pH-Responsive Tumor MR/Optical Imaging and Photothermal Therapy. Small.

[345] Tan R.Y.H., Lee C.S., Pichika M.R., Cheng S.F., Lam K.Y. (2022). PH Responsive Polyurethane for the Advancement of Biomedical and Drug Delivery. Polymers.

[346] Zhu Y.J., Chen F. (2015). pH-responsive drug-delivery systems. Chem. Asian J..

[347] Yu H., Jin F., Liu D., Shu G., Wang X., Qi J., Sun M., Yang P., Jiang S., Ying X. (2020). ROS-responsive nano-drug delivery system combining mitochondria-targeting ceria nanoparticles with atorvastatin for acute kidney injury. Theranostics.

[348] Guo W., Chen Z., Feng X., Shen G., Huang H., Liang Y., Zhao B., Li G., Hu Y. (2021). Graphene oxide (GO)-based nanosheets with combined chemo/photothermal/photodynamic therapy to overcome gastric cancer (GC) paclitaxel resistance by reducing mitochondria-derived adenosine-triphosphate (ATP). J. Nanobiotechnol..

[349] Khakpour E., Salehi S., Naghib S.M., Ghorbanzadeh S., Zhang W. (2023). Graphene-based nanomaterials for stimuli-sensitive controlled delivery of therapeutic molecules. Front. Bioeng. Biotechnol..

[350] Yaghoubi F., Motlagh N.S.H., Naghib S.M., Haghiralsadat F., Jaliani H.Z., Moradi A. (2022). A functionalized graphene oxide with improved cytocompatibility for stimuli-responsive co-delivery of curcumin and doxorubicin in cancer treatment. Sci. Rep..

[351] Ballance W.C., Qin E.C., Chung H.J., Gillette M.U., Kong H. (2019). Reactive oxygen species-responsive drug delivery systems for the treatment of neurodegenerative diseases. Biomaterials.

[352] Liang J., Liu B. (2016). ROS-responsive drug delivery systems. Bioeng. Transl. Med..

[353] Rinaldi A., Caraffi R., Grazioli M.V., Oddone N., Giardino L., Tosi G., Vandelli M.A., Calza L., Ruozi B., Duskey J.T. (2022). Applications of the ROS-Responsive Thioketal Linker for the Production of Smart Nanomedicines. Polymers.

[354] Oddone N., Boury F., Garcion E., Grabrucker A.M., Martinez M.C., Da Ros F., Janaszewska A., Forni F., Vandelli M.A., Tosi G. (2020). Synthesis, Characterization, and In Vitro Studies of an Reactive Oxygen Species (ROS)-Responsive Methoxy Polyethylene Glycol-Thioketal-Melphalan Prodrug for Glioblastoma Treatment. Front. Pharmacol..

[355] Shen C., Gao M., Chen H., Zhan Y., Lan Q., Li Z., Xiong W., Qin Z., Zheng L., Zhao J. (2021). Reactive oxygen species (ROS)-responsive nanoprobe for bioimaging and targeting therapy of osteoarthritis. J. Nanobiotechnol..

[356] Zambonino M.C., Quizhpe E.M., Mouheb L., Rahman A., Agathos S.N., Dahoumane S.A. (2023). Biogenic Selenium Nanoparticles in Biomedical Sciences: Properties, Current Trends, Novel Opportunities and Emerging Challenges in Theranostic Nanomedicine. Nanomaterials.

[357] Cao W., Gu Y., Li T., Xu H. (2015). Ultra-sensitive ROS-responsive tellurium-containing polymers. Chem. Commun..

[358] Wang J., Sun X., Mao W., Sun W., Tang J., Sui M., Shen Y., Gu Z. (2013). Tumor redox heterogeneity-responsive prodrug nanocapsules for cancer chemotherapy. Adv. Mater..

[359] Li W., Li M., Qi J. (2021). Nano-Drug Design Based on the Physiological Properties of Glutathione. Molecules.

[360] Liping Y., Jian H., Zhenchao T., Yan Z., Jing Y., Yangyang Z., Jing G., Liting Q. (2022). GSH-responsive poly-resveratrol based nanoparticles for effective drug delivery and reversing multidrug resistance. Drug Deliv..

[361] Wilson D.S., Dalmasso G., Wang L., Sitaraman S.V., Merlin D., Murthy N. (2010). Orally delivered thioketal nanoparticles loaded with TNF-alpha-siRNA target inflammation and inhibit gene expression in the intestines. Nat. Mater..

[362] Sun C.Y., Cao Z., Zhang X.J., Sun R., Yu C.S., Yang X. (2018). Cascade-amplifying synergistic effects of chemo-photodynamic therapy using ROS-responsive polymeric nanocarriers. Theranostics.

[363] Yue C., Yang Y., Zhang C., Alfranca G., Cheng S., Ma L., Liu Y., Zhi X., Ni J., Jiang W. (2016). ROS-Responsive Mitochondria-Targeting Blended Nanoparticles: Chemo- and Photodynamic Synergistic Therapy for Lung Cancer with On-Demand Drug Release upon Irradiation with a Single Light Source. Theranostics.

[364] Kang C., Cho W., Park M., Kim J., Park S., Shin D., Song C., Lee D. (2016). H_2_O_2_-triggered bubble generating antioxidant polymeric nanoparticles as ischemia/reperfusion targeted nanotheranostics. Biomaterials.

